# Organic Sulfur Markers as Proxies of Depositional Paleoeenvironments Related to Recôncavo and Amazon Basins, Brazil

**DOI:** 10.1021/acsomega.4c07344

**Published:** 2024-11-01

**Authors:** Diego Nery do Amaral, Flávia Lima e Cima Miranda, Lua Morena Leôncio de Oliveira, José Roberto Cerqueira, Hélio Jorge Portugal Severiano Ribeiro, Olívia Maria Cordeiro Oliveira, Antônio Fernando de Souza Queiroz, Sérgio Luís Costa Ferreira, Maria Elisabete Machado

**Affiliations:** † Programa de Pós-Graduação em Petróleo e Meio Ambiente, Universidade Federal da Bahia, 40170-115 Salvador, BA, Brazil; ‡ Instituto de Química, Universidade Federal da Bahia, 40170-115 Salvador, BA, Brazil; § Instituto Nacional de Energia e Ambiente, INCT&EIA, 40170-115 Salvador, BA, Brazil; ∥ Departamento de Ciências Exatas, Universidade Estadual de Feira de Santana, 44036-900 Feira de Santana, BA, Brazil; ⊥ Laboratório de Engenharia e Exploração de Petróleo, LENEP, 27930-480 Macaé, RJ, Brazil

## Abstract

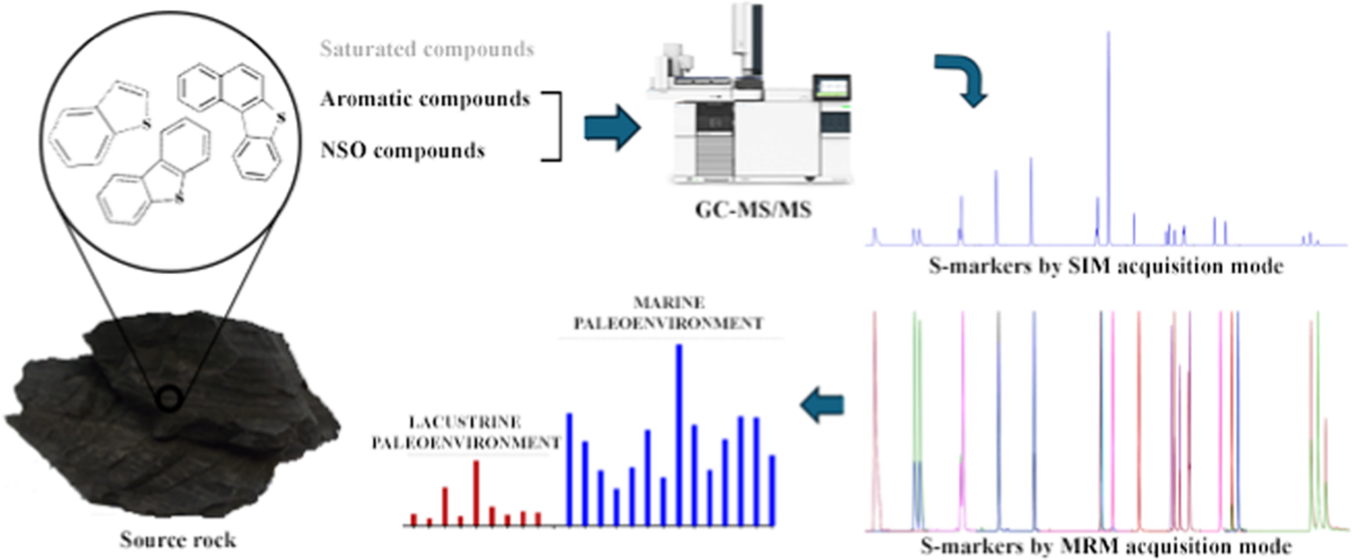

This study employed organic sulfur markers (S-markers) associated with geochemistry parameters to evaluate the paleoenvironment of different depositional settings in 24 samples collected in vertical sections of outcrops of the Candeias and Barreirinha Formations in Recôncavo and Amazon basins, respectively. A total of twenty-one S-markers from benzothiophene (BT), dibenzothiophene (DBT), and benzonaphtothiophenes (BNT) classes were optimized and quantified by gas chromatography-triple quadrupole mass spectrometry (GC–MS/MS). S-markers efficiently evaluated and differentiated the depositional paleoenvironment in the source rocks based on the individual compound, in cross-validation with saturated biomarkers, and associated with parameters such as total organic carbon (TOC) and Rock-Eval pyrolysis. Samples from the lacustrine environment presented low concentrations of BT, DBT, and BNT, and samples from the marine environment showed high BT, DBT, and BNT concentrations. The variations in ∑DBT and TOC indicated that the quantity and/or the type of organic matter exert some control over the distribution of DBTs. Although the formations are from different paleoenvironments, the organic matter input was similar, as indicated by high proportions of 1,2-BNT and 2,1-BNT relative to 2,3-BNT, thus characterizing the algal input with a microbial contribution for both sites. The sum of the BNTs was directly related to the amounts of amorphous organic matter (AOM) in the vertical distribution of outcrops. These results are in accordance with the finding that BNTs may originate from the microbial activity. The DBT/Phen vs pristane/phytane (Pr/Ph) relationship attested to differences in the redox conditions of the depositional paleoenvironments of the formations under study. The 4,6-DMDBT/2,4,6-TMDBT and 2,4,6-TMDBT/(2,4,7 + 2,4,8)-TMDBT ratios indicated immaturity for hydrocarbon generation.

## 1 Introduction

Sulfur is an abundant heteroatom in fossil fuels with total concentrations usually less than 4.0%. The polycyclic aromatic sulfur heterocycle (PASH) structures are the most abundant form of organic sulfur in crude oils and source rocks and include benzothiophenes (BTs), dibenzothiophenes (DBTs), benzonaphthothiophenes (BNTs), and their C_1_–C_3_ alkyl derivatives.^1^ In geochemistry studies, the PASHs have been used as markers (S-markers) to assess the depositional paleoenvironment, maturity degree, migration, and organic facies from crude oils and source rock extracts.^1−3^

S-markers provide a convenient way to correlate oils with source rocks, to assess facies, organic matter input, and depositional paleoenvironment conditions. For example, the abundance of dibenzothiophenes (e.g., DBT and methyl-DBTs) in oils from different sedimentary environments increases in the order of freshwater < saline < hypersaline facies.^4^ For the benzonaphthothiophenes and their isomers (C_1_-BNTs, C_2_-BNTs, and C_3_-BNTs), the abundances in crude oils and source rocks of terrigenous origin are lower than marine equivalents.^4−7^ In addition, due to their stability in high temperatures, S-markers can be ideally used to assess the state of maturity.^8,9^ However, the studies based on S-markers to assess paleodepositional settings are limited.^2,10−12^

Saturated hydrocarbons, such as *n*-alkanes, pristane, phytane, hopanes, and steranes, are biomarkers conventionally employed in geochemistry studies to provide information about the origin and depositional paleoenvironment of the organic matter.^13^ These compounds determine the relationship between crude oil and residual organic matter in source rocks all over the world. In Brazil, the oils and source rocks from the Recôncavo and Amazon basins have already been characterized in terms of their paleoenvironment using different biomarkers.^14−18^ However, biomarkers may be affected by biodegradation, water washing, and thermal alteration.^19,20^ In these cases where parameters are not sufficiently diagnostic, S-markers can provide a better application.^7,21^ Therefore, the combined information based on the cross-validation of saturated biomarkers and S-markers allows a more accurate evaluation of source rocks’ depositional paleoenvironments.

In the Amazon Basin, the Barreirinha Formation is known as the primary hydrocarbon source rock composed of dark gray to black shale. Data from saturated biomarkers indicate that these black shales, highly enriched in organic matter, were deposited in a marine paleoenvironment.^22,23^ In the Recôncavo basin, as biomarker data indicate, the Candeias Formation is recognized as a hydrocarbon source rock deposited in a lake context with good organic matter preservation.^14,15,24^ Considering that the two sedimentary basins were deposited in different paleoenvironments (marine and lacustrine), a detailed study based on specific S-markers using these basins as a model can be an excellent tool for paleodepositional assessment.

Determination of S-markers in crude oils is made primarily by gas chromatography coupled to mass spectrometry (GC–MS) in the selected ion monitoring (SIM) mode.^1^ However, it is necessary to perform previous laborious fractionation steps, and there are limitations to the identification of compounds with the same mass fragment ions. The gas chromatography coupled to triple quadrupole spectroscopy (GC–MS/MS) is able to increase the selectivity and sensitivity for S-markers because it eliminates the background interference. The GC–MS/MS has been proven to good performance in the analysis of individual S-markers in petroleum samples.^25−29^ However, a limited number of compounds was evaluated, and the geochemistry interpretation of results was not the aim of those studies.

This work aimed to employ S-markers in combination with geochemistry parameters to assess the depositional paleoenvironment of source rocks of different origins. A GC–MS/MS method was optimized to determine twenty-one S-markers in source rock extracts. Interpretations focused on individual and diagnostic ratios from S-markers were used, for the first time, to analyze differences and similarities between the geological formations under study and to interpret the paleoenvironment conditions of their depositional settings.

## 2 Geological Settings

### 2.1 Amazon Basin

**Figure 1:**
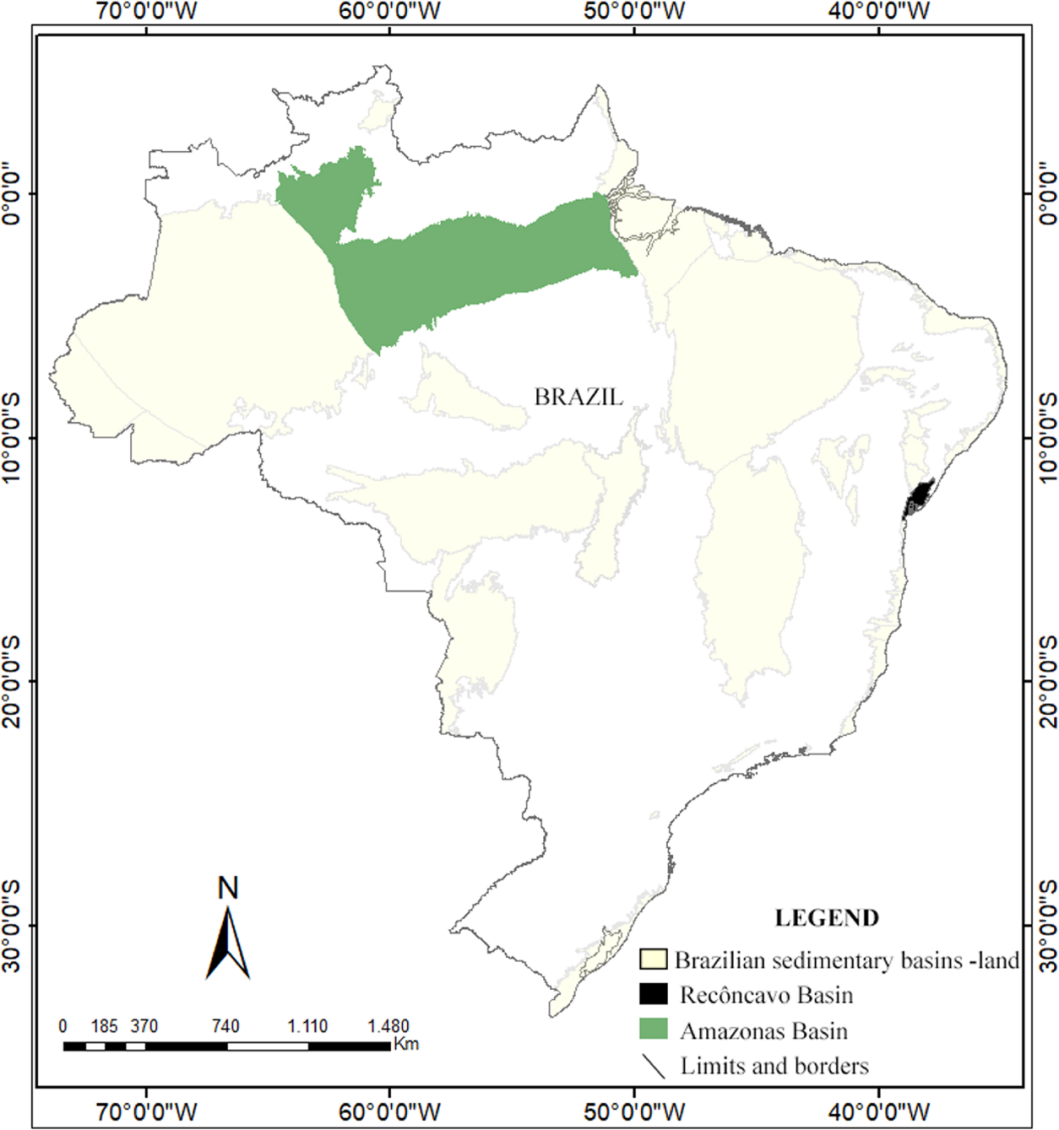
Map of Brazilian terrestrial sedimentary basins highlighting the Recôncavo and Amazon basins.

**Figure 2:**
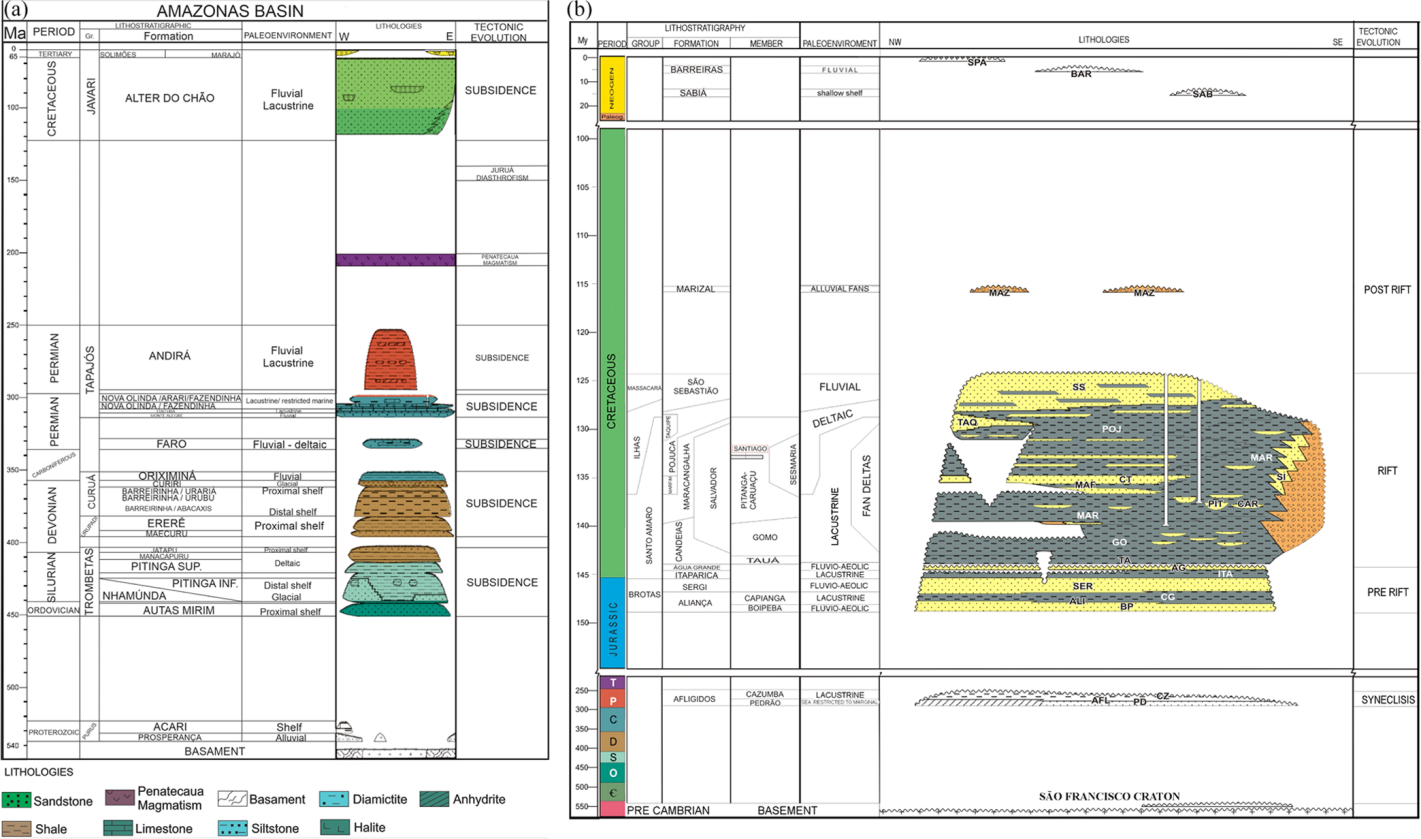
Stratigraphic charts: (a) Amazon basin. (b) Recôncavo basin, adapted from refs (17,34).

The Amazon basin is located in the northern region of Brazil (Figure 1), covering part of the states of Amazon and Pará, with an area of approximately 500,000 km^2^. It is classified as a Paleozoic basin of the intracratonic syneclisis type, whose sedimentation began in the Paleozoic and lasted until the Mesozoic,^16,17^ as seen on the stratigraphic chart of the basin (Figure 2a).

The deposition of the Barreirinha Formation is associated with a rapid relative sea level rise that occurred when the South American Platform underwent a major marine transgression in Frasnian.^30^ The initial depositional phase of this formation is represented by a thick section of radioactive black shales (dark gray, laminated, fissile, and bituminous) denoted as the abacaxis member, which is considered the primary hydrocarbon source rock in the Amazon basin. The abacaxis member is overlapped by two other members: Urubu (dark gray shales) and Uraria (gray shales and dark to light siltstones).^30^ Previous studies indicate that the Barreirinha Formation is the carrier of marine organic matter, with average TOC contents between 2 and 3%, mostly type II kerogen, and varying levels of thermal maturation.^16,23^

### 2.2 Recôncavo Basin

The Recôncavo basin covers approximately 11,500 km^2^ from Bahia state in Brazil (Figure 1) and corresponds to the southern portion of the Recôncavo-Tucano-Jatobá Rift (RTJ). The RTJ system developed in the Cretaceous can be interpreted as an aulacogen segment associated with South Atlantic Rift.^31^

The deposition of the Candeias Formation, recognized as source rocks in the Recôncavo basin, occurred with increased tectonic activity added to the predominance of climate humidification, generating conditions for the development of deep lakes, which marked the beginning of the rift phase in the Berriasian (Figure 2b). In this scenario, initially there was the transgression generating the dark pelite of the Tauá Member, which is overlapped by gray-green shale with carbonatic intercalation of the Gomo Member.^32^ Various geochemical evaluation studies in Candeias Formation have been performed, including the identification of strata with multiple amounts of total organic carbon (TOC), suggesting internal faciological changes in this geological formation.^14,15,24,33^

Thus, the Candeias Formation is defined as a thick section of dark-green, gray shales, with subordinate intersperses of limestone and dolomites, locally encompassing bodies of massive and/or stratified sandstones.^24,34^ According to Amaral et al.^15^ the Candeias shales (Gomo Member) are bearers of amorphous organic matter (AOM) with intense fluorescence, average total organic carbon (TOC) content of approximately 3% and kerogen type I, with a high potential for hydrocarbon generation.

## 3 Materials and Methods

### 3.1 Sampling

**Figure 3:**
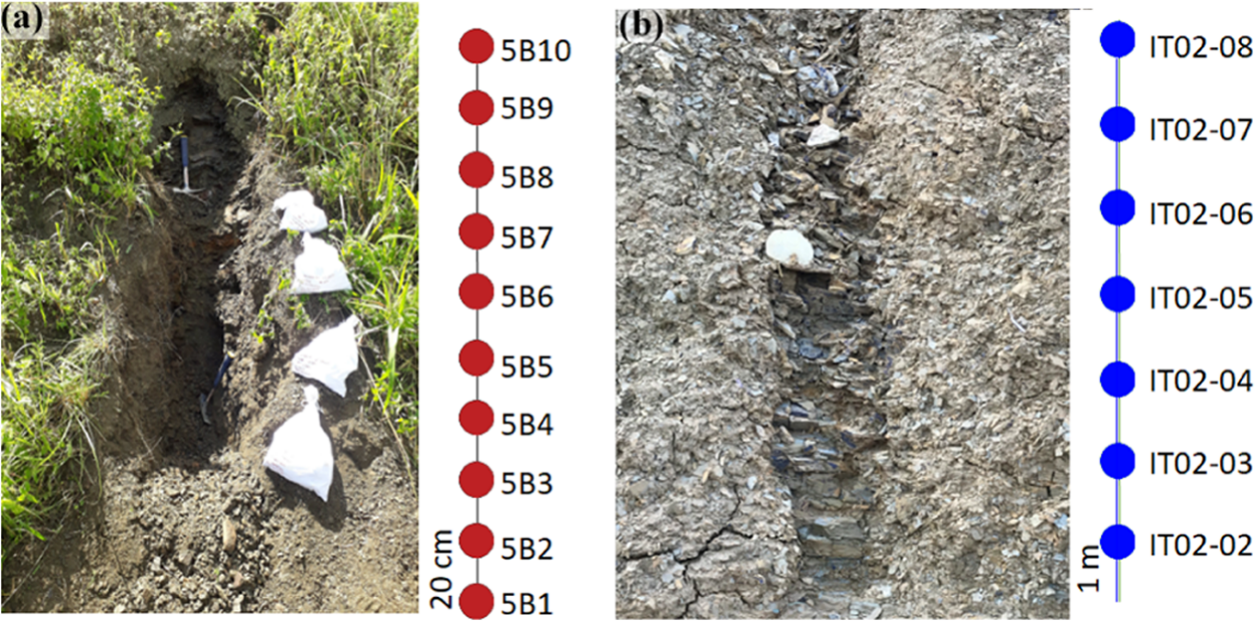
Sampling points with vertical spacing in outcrops of the Candeias (a) and Barreirinha (b) formations.

Samples in the Recôncavo basin were collected from an outcrop on the side of highway BR 324, km 557, in the municipality of Santo Amaro, Bahia, Brazil. The sampling was carried out every 20 cm for a total of 10 samples (labeled 5B1 to 5B10, Figure 3a), to investigate possible vertical variations of the geochemical parameters.

Two outcrops of the Amazon basin were selected, identified as IT02 and IT06 (representative image of the IT02 outcrop in Figure 3b), and both were collected vertically with a spacing of 1 m at different points (*n* = 14) along the BR 230 highway in the Rurópolis municipality, Pará state, Brazil. Figure 3 shows the sampling in the Recôncavo basin (Figure 3a) and a representative outcrop of the Amazon basin (Figure 3b). The collection procedure was the same for all of the outcrops. The initial layers of altered rocks were removed, digging deep enough (between 1 and 2 m) to reach below the weathering zone to access examples of source rocks without alteration/oxidation features.

### 3.2 Extraction of Soluble Organic Matter

Prior to extraction, the rock samples were ground with an agate mortar and pestle, pulverized in a Retsch planetary ball mill (Retsch, PM 400, Haan, Germany) and subsequently sieved through a steel mesh sieve with an opening of 0.180 mm (80 mesh), and stored in glass recipients.

Accelerated solvent extraction (Dionex ASE 350, Thermo Scientific, Massachusetts) was employed to obtain the soluble organic matter present in rock samples.^35^ Initially, 50 g of sample and 10 g of diatomaceous earth, a dehumidifying agent (Celite 545, Exodo Cientfica, Brazil), were added to metal extractor cells. Thus, the system was heated at 75 °C at a pressure of 5 × 10^6^ Pa for 15 min using 150 mL of dichloromethane. The procedure was repeated three times to ensure that all soluble organic matter compounds were extracted. Then, the source rock extract was concentrated (solvent evaporated) using a rotary evaporator (R-100, Buchi, Meierseggstrasse, Switzerland).

### 3.3 Fractionation of Soluble Organic Matter

The extracts were fractionated by open column chromatography using silica as the stationary phase (ASTM D2007-11).^36^ The saturated hydrocarbon fractions were eluted with 25 mL of *n*-hexane, and the aromatic hydrocarbon fractions containing the S-markers were eluted with 30 mL of *n*-hexane:DCM (4:1, v/v) and 30 mL of DCM:methanol (4:1, v/v). All fractions were concentrated in a rotary evaporator (Model R-210 Labortechnik, AG Switzerland) and transferred to 2 mL vials.

### 3.4 GC–MS/MS Analyses of S-Markers

The GC–MS/MS analyses of the S-markers were performed on an Agilent 7890B gas chromatograph equipped with a split/splitless injector, a DB5MS column (5% phenylmethylpolysiloxane, 30 m × 0.25 mm internal diameter ×0.25 μm film thickness) coupled to an Agilent 7000C mass spectrometer (Santa Clara, CA). The GC operating conditions are as follows: the oven temperature was held isothermally at 100 °C for 2 min, ramped to 310 °C at 5 °C min^–1^, and held isothermal for 1.5 min. Helium was used as the carrier gas with a constant flow rate of 1.0 mL min^–1^. The MS was operated in the electron ionization (EI) mode at 70 eV, ion source at 280 °C, and injector and transfer line temperature of 300 °C.^29^

#### 3.4.1 MS/MS Optimization

The MS/MS transitions for some BTs, DBTs, and BNTs were determined by Sampaio et al.^29^ However, in the present study, the number of compounds was twenty-one and tested the collision energy (CE) for each individual S-marker. Thus, to optimize the multiple reaction monitoring (MRM) conditions for the compounds used in this study, a full scan and product ion scan (PIS) were performed in MS/MS. Initially, the mass spectra of all individual standards were obtained in the full scan mode (*m*/*z* mass range 45–450), and the fragments with the highest abundance for each one were selected, observing their retention times. Windows were defined based on the retention times of the compounds of interest in the SIM mode. The PIS was selected using collision energy variation from 5 to 60 eV (5, 10, 20, 30, 40, 50, and 60 eV). The product ions exhibiting the highest sensitivity were selected as quantification ions, whereas those exhibiting the second highest sensitivity were selected as qualification ions. Thus, two transitions were defined in the MRM mode under optimized collision energies.

For the method optimization, the following individual standards were employed at a concentration of 100 ug L^–1^: benzothiophene (BT), 2-methylbenzothiophene (2-MBT), 3-methylbenzothiophene (3-MBT), 2,4-dimethylbenzothiophene (2,4-DMBT), 2,6-dimethylbenzothiophene (2,6-DMBT), 2,3,4-trimethylbenzothiophene (2,3,4-TMBT), 2,5,7-trimethylbenzothiophene (2,5,7-TMBT), dibenzothiophene (DBT), dibenzothiophene-*d*_8_ (DBT-*d*_8_), phenanthrene (Phen), 4-methyldibenzothiophene (4-MDBT), 1-methyldibenzothiophene (1-MDBT), 2,8-dimethyldibenzothiophene (2,8-DMDBT), 4,6-dimethyldibenzothiophene (4,6-DMDBT), 2,4-dimethyldibenzothiophene (2,4-DMDBT), 1,4-dimethyldibenzothiophene (1,4-DMDBT), 3,6/2,6-dimethyldibenzothiophene (3,6/2,6-DMDBT), 2,4,7-trimethyldibenzothiophene (2,4,7-TMDBT), 4,6-diethyldibenzothiophene (4,6-DEDBT), benzo[*b*]naphto[1,2-*d*]thiophene (BNT- 1,2), benzo[*b*]naphtho[2,1-*d*]thiophene (BNT-2,1), and benzo[*b*]naphtho[2,3-*d*]thiophene (BNT-2,3).

For BT, DBT-*d*_8_, 3-MBT, 2,4-DMBT, 2,3,4-TMBT, DBT, Phen, 4-MDBT, 1-MDBT, 4,6-DMDBT, 2,4-DMDBT, 1,4-DMDBT, 2,4,7-TMDBT, 4,6-DEDBT and BNT-1,2 the same MRM transitions defined by Sampaio et al.^29^ were employed. For 2-MBT, 2,6-DMBT, 2,5,7-TMBT, 2,8-DMDBT, 3,6/2,6-DMDBT, BNT-2,1 and BNT-2,3, the MS/MS conditions were optimized and definite.

### 3.5 Total Organic Carbon, Total Sulfur, and Rock-Eval Pyrolysis

The TOC content was determined from 1.0 g of each sample (80 mesh) subjected to acid digestion (HCl, 37%) to carbonate removal and measured using a LECO 628CN Elementary Analyzer. Total sulfur contents were performed on the LECO 628S Elementary Analyzer. Rock-Eval analysis was performed using a Rock-Eval 6 instrument according to the procedure proposed by Behar et al.^37^ The parameters included 100 mg of each sample (0.177 mm) added to a tin device. Analyses were previously performed by Amaral et al. and Góes et al.^15,23^

### 3.6 Biomarkers Analysis

Saturated biomarkers were previously analyzed in a gas chromatograph coupled to a mass spectrometer (GC/MS-DSM5977A, Agilent) using a DB-5MS capillary column (60 m × 0.25 mm × 0.25 μm). Helium was used as the carrier gas at a flow of 1 mL min^–1^. The samples were diluted in hexane at 0.05 mg for each 1 mL of solvent. The injection volume was 1 μL in the splitless mode. The oven temperature program was from 60 to 310 °C with a heating rate of 2 °C min^–1^. The following ions were monitored in MS: *m*/*z* 217 (steranes), *m*/*z* 191 (terpanes), and *m*/*z* 259 (tetracyclic polyprenoids and diasteranes).^15,23^

### 3.7 Palynofacies

The palynofacies analysis was carried out qualitatively and quantitatively^15,23^ by counting 300 organic components (amorphous organic matter, phytoclasts, and palynomorphs) on each slide using a Zeiss Axio Imager A2m microscope, equipped with a white (halogen) light source (from a 12 V/100 W halogen lamp with stabilized current) and a UV light (fluorescence) source (from a high-pressure 100 W mercury lamp with stabilized current). Counting was performed under 20 times magnification following the procedure proposed by Tyson.^60^ Qualitatively, organic matter was evaluated for the degree of preservation, appearance, color, presence, and intensity of fluorescence under excitation with UV/blue-violet light.

## 4 Results and Discussion

### 4.1 Identification and Quantification of S-Markers by GC–MS/MS

**Figure 4:**
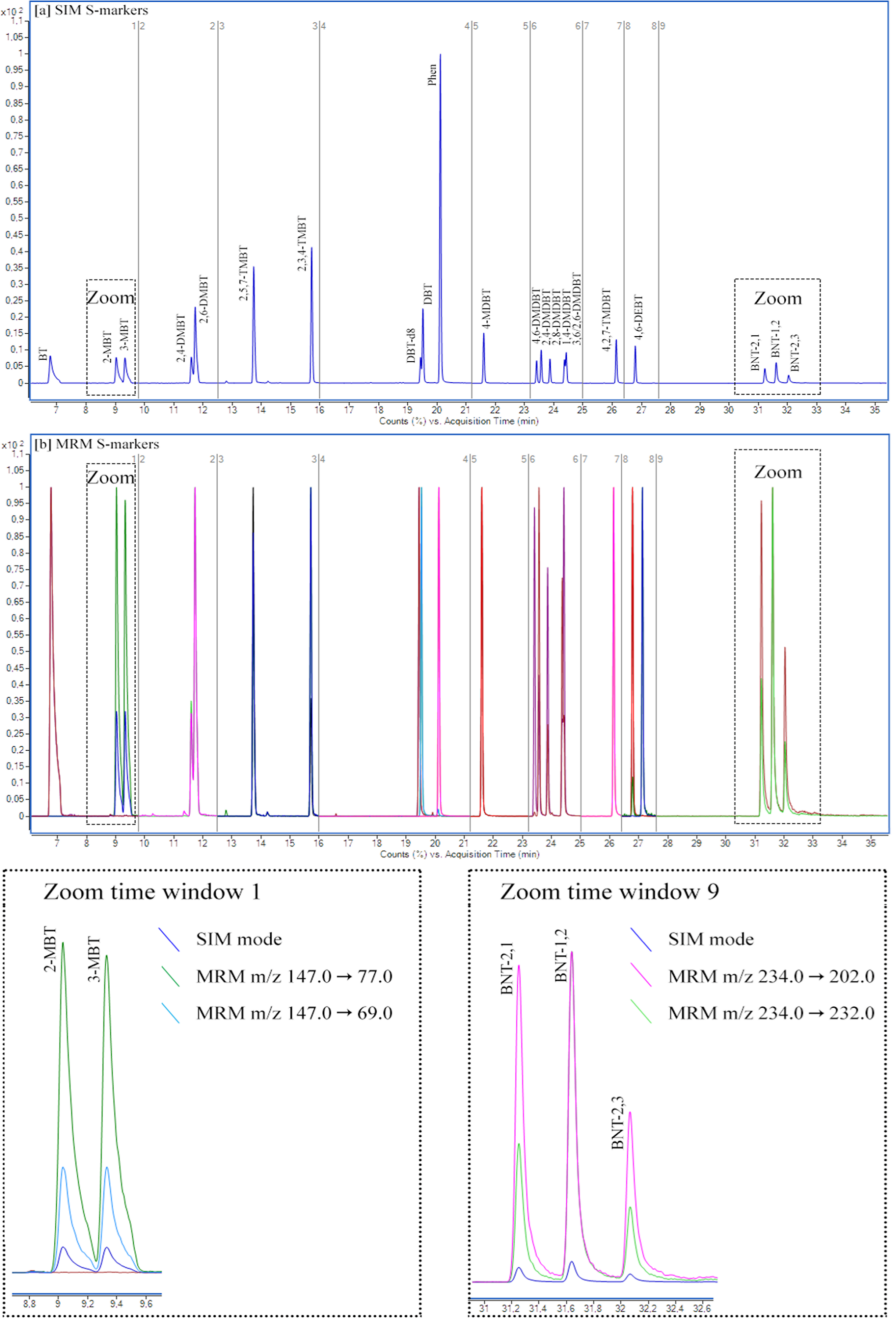
Chromatograms of S-markers in (a) SIM mode and (b) MRM mode. Different colors in (b) and (c) refer to individual MRM transitions.

The critical parameters for the identification and quantification of compounds in the GC–MS/MS method include the choice of the precursor ion and the product ion for each MRM transition with specific collision energy (CE). The more intense ion in the mass spectrum was chosen as the precursor ion, and the two fragment ions with the highest response were selected as the product ion. For example, *m*/*z* 147 → 77 and 147 → 69 were used for MBTs (Sampaio et al.,^29^) and substantially reduced the baseline level with the signal-to-noise ratio increased approximately 10 times when compared to GC–MS (*m*/*z* 147) (Figure 4).

The optimized GC–MS/MS conditions for the analysis of 21 standards of S-markers are summarized in Table 1, including the retention times (*R*_t_), window widths, quantification and confirmation transitions, and collision energies (CE).

**Table 1:** Summary of Optimized MRM Transitions Used for Quantifying S-Marker Compounds

	window	*R*_t_	quantification transition			confirmation transition		
S-markers	(±*R*_t_ min)	(min)	precursor ion (*m*/*z*)	product ion (*m*/*z*)	CE^b^ (eV)	precursor ion (*m*/*z*)	product ion (*m*/*z*)	CE^b^ (eV)
BT	4.00–10.00	6.77	134	89	20	134	69	20
2-MBT^a^	4.00–10.00	9.03	147	77	30	147	69	30
3-MBT	4.00–10.00	9.32	147	77	40	147	69	30
2,4-DMBT	10.00–12.00	11.60	162	161	20	162	128	40
2,6-DMBT^a^	10.00–12.00	11.73	162	161	20	162	147	30
2,5,7-TMBT^a^	12.00–17.00	13.73	176	161	20	176	128	40
2,3,4-TMBT	12.00–17.00	15.71	176	161	20	176	175	40
DBT-*d*_8_	17.00–21.00	19.44	192	146	30	192	160	40
DBT	17.00–21.00	19.52	184	139	30	184	152	20
Phen	17.00–21.00	20.12	178	176	30	178	152	40
4-MDBT	21.00–23.00	21.60	198	197	30	198	165	30
4,6-DMDBT	23.00–25.00	23.41	212	197	20	212	211	40
2,4-DMDBT	23.00–25.00	23.57	212	211	20	212	197	40
2,8-DMDBT^a^	23.00–25.00	23.87	212	197	40	212	211	50
1,4-DMDBT	23.00–25.00	24.37	212	211	20	212	197	40
3,6/2,6-DMDBT^a^	23.00–25.00	24.48	212	197	30	212	211	30
2,4,7-TMDBT	25.00–30.00	26.13	226	211	20	226	225	40
4,6-DEDBT	25.00–30.00	26.79	240	210	20	240	225	40
BNT-2,1^a^	30.00–45.50	31.22	234	202	30	234	189	40
BNT-1,2	30.00–45.50	31.61	234	202	40	234	189	30
BNT-2,3^a^	30.00–45.50	32.04	234	202	30	234	189	40

a Compounds optimized in this study.

b Collision energy.

The concentration of S-markers obtained from source rock extracts by optimized conditions of GC–MS/MS are shown in Table 2. In general, higher concentrations were found in samples from the Barreirinha Formation. The 2,6-DMBT was the compound with the lowest concentration (nd to 0.01 μg g^–1^ for 5B-06), while 2 + 3MDBT showed the highest concentration (1938 μg g^–1^ to IT06-59).

**Table 2:** Concentrations of Sulfur Markers Applied to This Study (μg g^–1^ of Extract) in Samples from the Recôncavo (5B) and Amazon Basins (IT)^a^

Recôncavo basin										
S-marker	5B-01	5B-02	5B-03	5B-04	5B-05	5B-06	5B-07	5B-08	5B-09	5B-10
BT	0.08	0.02	0.03	0.75	0.35	0.16	0.37	0.41	0.16	1.26
2-MBT	0.04	0.01	nd	0.02	0.01	0.10	0.01	0.01	nd	0.02
3-MBT	0.04	0.01	0.01	0.02	0.05	0.14	0.01	0.01	nd	nd
2,4-DMBT	0.01	nd	nd	0.01	0.01	0.01	0.01	0.01	nd	0.01
2,6-DMBT	nd	nd	nd	nd	nd	0.01	nd	nd	nd	nd
2,5,7-TMBT	0.15	0.04	0.06	0.10	0.26	0.24	0.08	0.18	0.01	0.09
2,3,4-TMBT	0.01	nd	nd	0.01	0.01	nd	nd	nd	nd	nd
DBT	0.10	0.08	0.33	0.20	0.18	0.24	0.24	0.25	0.17	0.23
Phen	0.64	0.92	5.83	0.95	0.82	1.30	0.57	0.43	0.28	0.36
1-MDBT	0.18	0.15	0.64	0.23	0.23	0.37	0.19	0.17	0.13	0.26
(2 + 3)-MDBT	0.15	0.07	0.39	0.15	0.13	0.22	0.12	0.05	0.07	0.10
4-MDBT	0.22	0.16	0.62	0.34	0.30	0.58	0.25	0.22	0.17	0.19
4,6-DMBT	0.05	0.03	0.08	0.05	0.06	0.07	0.05	0.05	0.04	0.13
2,3-DMBT	0.05	0.04	0.03	0.07	0.04	0.02	0.05	0.05	0.12	0.20
2,4-DMBT	0.05	0.05	0.19	0.07	0.05	0.19	0.04	0.02	0.03	0.02
2,8-DMBT	0.04	0.02	0.07	0.04	0.06	0.06	0.04	0.05	0.04	0.05
1,4-DMBT	0.02	0.01	0.01	0.04	0.03	0.01	0.02	0.02	0.01	0.05
3,6/2,6-DMBT	0.03	nd	0.04	0.04	0.03	0.15	0.06	0.04	0.02	0.03
2,4,6-TMDBT	0.10	0.04	0.24	0.19	0.16	0.25	0.20	0.17	0.23	0.25
2,4,7-TMDBT	0.13	0.06	0.18	0.19	0.18	0.17	0.15	0.14	0.14	0.23
2,4,8-TMDBT	0.10	0.04	0.18	0.14	0.12	0.18	0.11	0.10	0.10	0.16
4,6-DEBT	nd	nd	nd	0.01	nd	nd	nd	nd	nd	nd
BNT-2,1	0.79	0.39	0.55	2.07	1.85	0.89	1.25	2.22	3.01	3.62
BNT-1,2	0.02	0.01	0.02	0.05	0.05	0.02	0.03	0.04	0.02	0.08
BNT-2,3	0.02	0.01	0.01	0.07	0.08	0.06	0.09	0.14	0.13	0.16

Amazon basin														
S-marker	IT02-02	IT02-03	IT02-04	IT02-05	IT02-06	IT02-07	IT02-08	IT06-54	IT06-55	IT06-56	IT06-57	IT06-58	IT06-59	IT06-60
BT	7.18	0.39	5.44	4.44	3.08	14.20	5.09	10.90	11.43	11.83	11.54	9.43	15.54	10.46
2-MBT	0.47	0.07	7.89	3.85	1.90	2.34	6.07	2.87	3.78	4.48	3.88	3.39	2.56	4.76
3-MBT	4.23	0.45	34.94	11.43	8.89	17.98	23.43	41.42	30.55	20.02	26.94	29.68	22.19	26.98
2,4-DMBT	0.40	0.22	0.91	1.66	1.81	8.94	3.04	0.52	0.69	0.79	0.46	0.28	3.50	0.58
2,6-DMBT	0.27	0.08	0.79	0.71	0.53	0.87	0.71	0.56	0.59	0.48	0.56	0.18	0.71	0.30
2,5,7-TMBT	46.79	8.12	198	205.7	217	73.73	49.82	89.22	89.43	105	90.69	41.18	113	53.33
2,3,4-TMBT	4.37	0.69	9.50	9.89	10.04	14.34	10.53	25.77	22.56	21.36	15.96	5.72	23.19	13.88
DBT	306	60.25	631	637	639	845	892	1135	1061	700	759	369	1103	728
Phen	2133	168	2779	2620	2474	3147	2358	2626	2384	2293	2261	1269	2891	2398
1-MDBT	979	84.95	1088	1139	1197	1680	1490	1646	1334	1248	1135	1117	1486	1291
(2 + 3)-MDBT	1022	89.28	1160	1200	1294	1649	1601	1938	1675	1644	1441	1245	1665	1333
4-MDBT	867	80.65	987	1007	1100	1603	1349	1602	1441	1372	1317	1103	1220	1207
4,6-DMBT	91.31	7.91	91.51	101	107	166	118	154	134	124	124	121	147	128
2,3-DMBT	30.73	2.70	36.29	47.43	49.46	40.81	22.70	32.32	40.29	41.64	38.44	27.51	61.03	32.15
2,4-DMBT	89.68	6.07	73.95	81.96	87.05	139	117	129	120	106	101	91.13	98.32	97.01
2,8-DMBT	57.45	1.99	30.50	41.47	68.29	105	90.48	110	95.64	74.76	78.37	67.61	84.67	76.03
1,4-DMBT	52.76	3.16	50.64	51.96	56.62	81.09	68.49	84.58	76.56	67.50	67.64	67.52	79.68	56.07
3,6/2,6-DMBT	213	12.39	164	179	222	317	271	316	314	257	237	225.8	255	217
2,4,6-TMDBT	71.55	4.42	57.34	59.64	68.78	105	19.84	112	87.86	74.52	75.93	77.77	82.04	85.13
2,4,7-TMDBT	102	6.51	81.16	86.86	107	155	21.99	144	136	106	102	112	127	124
2,4,8-TMDBT	106	6.12	73.82	87.96	94.36	153	39.65	150	134	108	99.65	96.93	109	110
4,6-DEBT	1.50	0.10	1.24	1.36	1.63	2.39	1.97	2.49	2.25	1.82	1.74	1.64	1.71	1.65
BNT-2,1	77.36	4.54	71.74	72.06	73.41	78.23	61.43	92.43	91.14	90.44	90.39	85.40	82.26	77.90
BNT-1,2	44.92	2.44	38.40	44.43	47.01	61.96	45.35	55.72	53.44	52.71	49.61	46.63	48.66	48.20
BNT-2,3	18.45	1.21	21.39	26.43	27.78	30.49	20.10	25.21	23.46	19.86	26.90	30.22	28.05	23.19

a nd = not detected.

Throughout the literature, it is uncommon to find geochemistry studies aimed at the quantification of individual S-markers by GC–MS.^38−41^ The results are mainly expressed as the sum of classes^10,7,12,42,43^ or in area percentage values^3,25,44^ because of the commercial limitations and the high cost associated with acquiring the standards. However, GC–MS is not sufficiently selective for S-markers since coelutions with matrix compounds with the same *m*/*z* might interfere.^1,45^

**Figure 5:**
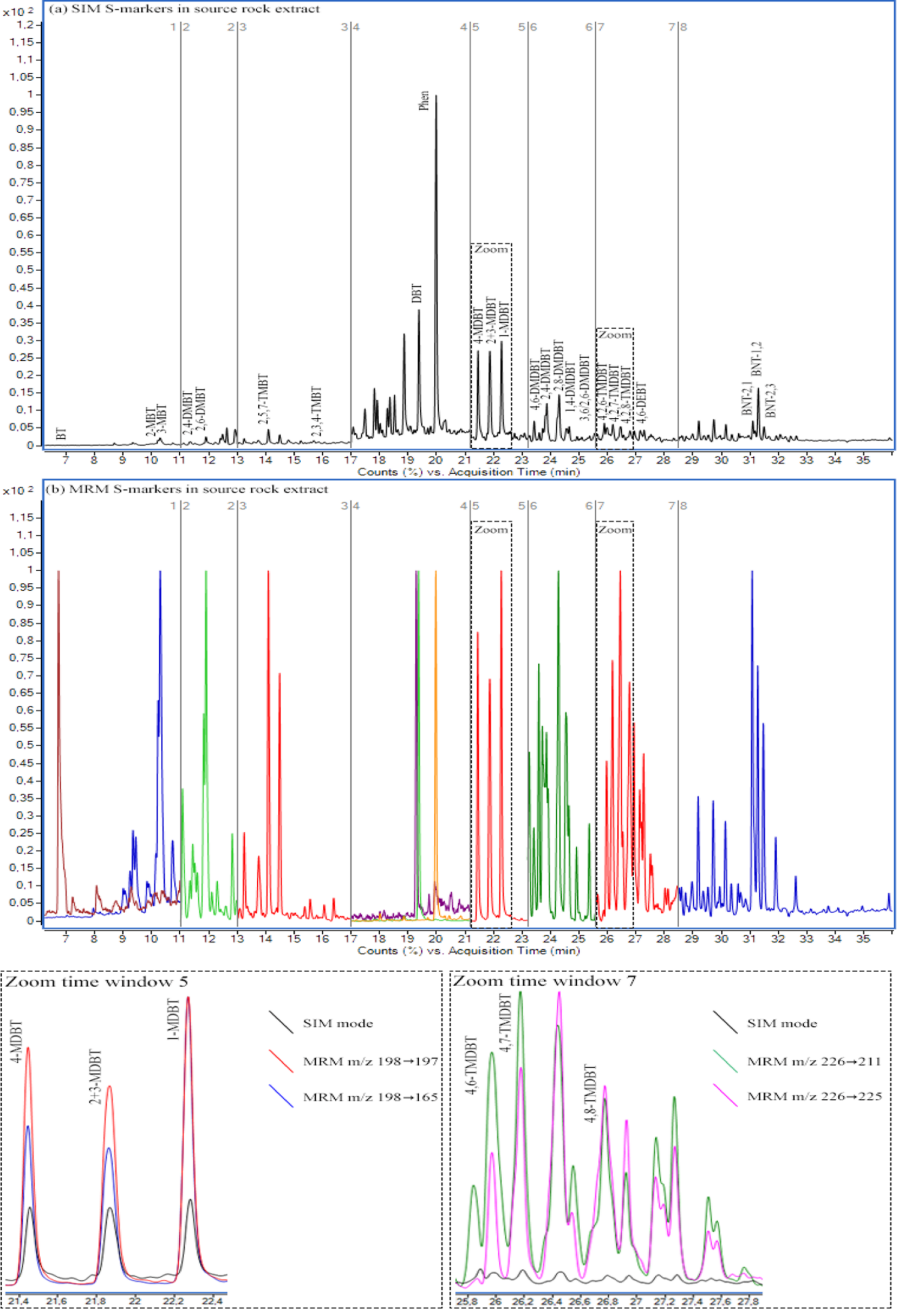
GC–MS/MS chromatogram of S-markers in source rock extract by the (a) SIM and (b) MRM mode.

A representative chromatogram of a source rock extract sample is shown in Figure 5. The triple quadrupole analyzer is considered one of the best alternatives to analyze S-markers.^1,29^ It minimizes interference by improving the selectivity based on the selection of appropriate precursor and product ions (Figure 5a). In addition, a significant decrease of chemical noise in the chromatogram is obtained when compared to that in the SIM mode (Figure 5b). Thus, thanks to the improved sensitivity, performing reliable determination of S-markers at trace levels, as those required in geochemistry studies is feasible.

### 4.2 Paleoenvironment Interpretation Based on S-Markers

#### 4.2.1 Origin of Organic Matter

The concentrations of BT, DBT, BNT, and its alkylated homologues in source rock extracts and crude oils have been employed to evaluate organic matter origin.^44,46^ The highest concentrations of these compounds are found in samples of marine origin compared to those of continental origin.^12,47^

**Figure 6:**
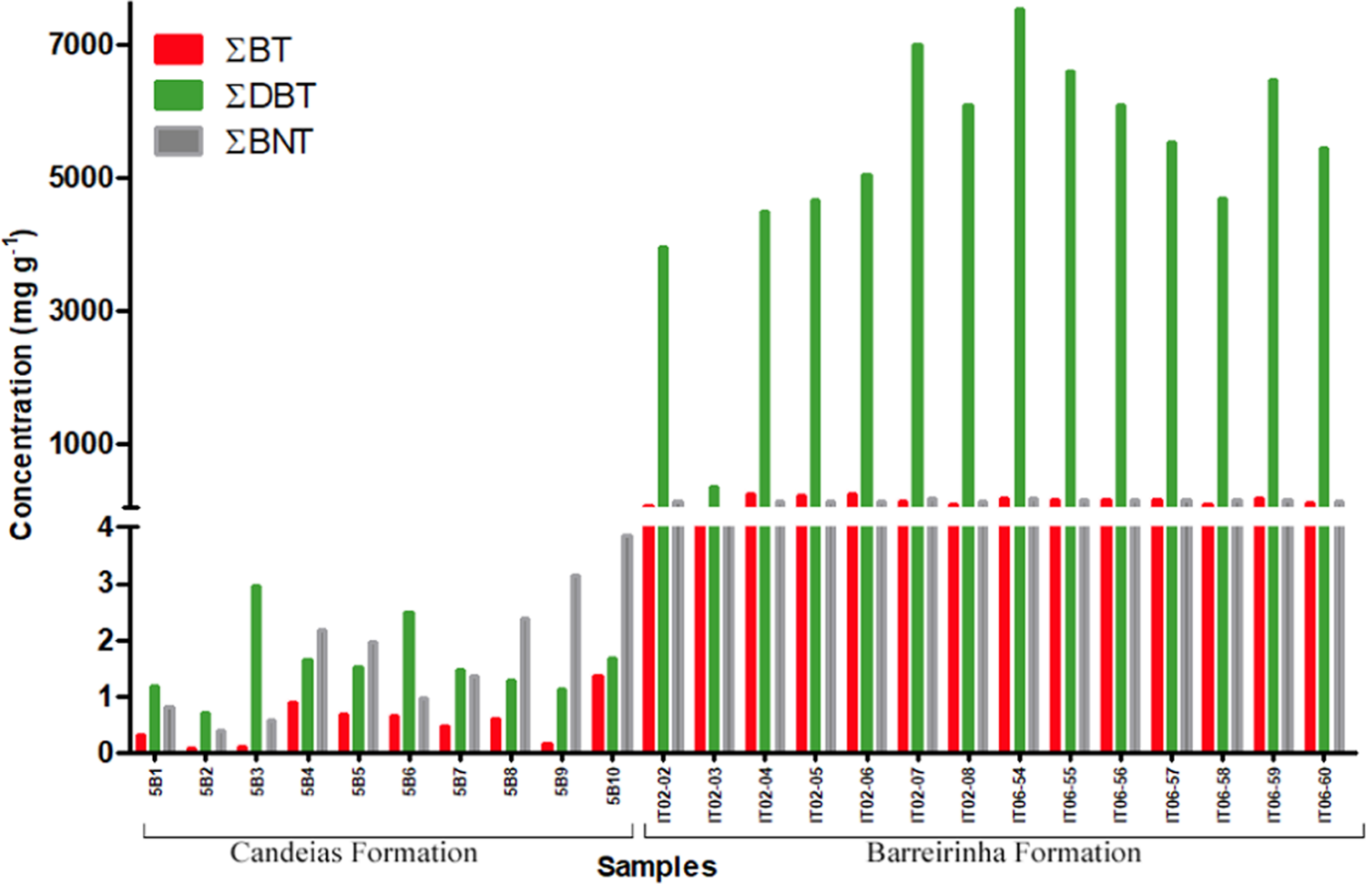
Histogram of the concentrations of benzothiophenes (∑BT), dibenzothiophenes (∑DBT), and benzonaphthothiophenes (∑BNT) in the samples.

The concentrations of individual S-markers in samples from the Recôncavo and Amazon basins were different (Table 2 and Figure 6). High concentrations of BT, DBT, and BNT, consistent with a marine paleoenvironment, were observed in the Barreirinha Formation. In contrast, low concentrations of these compounds were found in the Candeias Formation, which are typical of a continental freshwater paleoenvironment.

The differentiation of sedimentary paleoenvironments obtained by S-markers was confirmed by biomarkers, such as hopane/sterane (HOP/STE) (Figures S1–S4) and polyprenoids and diasteranes (TPP/TPP + DIA) ratios (Table S1). The palynofacies based on the relative proportions between amorphous organic matter (AOM), palynomorphs, and phytoclasts (Table S1) also confirmed the S-markers data. HOP/STE ratios greater than 5 and the presence of *Botryococcus* algae indicated that the organic matter present in the Candeias Formation had a lacustrine origin.^15^ In addition, values of HOP/STE were lower than 5, and the types of palynomorph found indicate the Barreirinha Formation’s marine origin.^23^

Another evaluation of the origin of organic matter based on S-markers is by DBTs, where the highest concentrations of these compounds are present in oils and extracts from source rocks of marine origin, compared to those of freshwater lacustrine origin.^10^ The classical biomarkers such as HOP/STE and TPP/[TPP + DIA] ratios are frequently employed to evaluate origin, where lake environments usually present values greater than 5 for the HOP/STE ratio and greater than 0.4 for the TPP/[TPP + DIA], while marine environments generally present values lower than 5 for the HOP/STE ratio and lower than 0.4 for the TPP/[TPP + DIA] ratio.^13^

**Figure 7:**
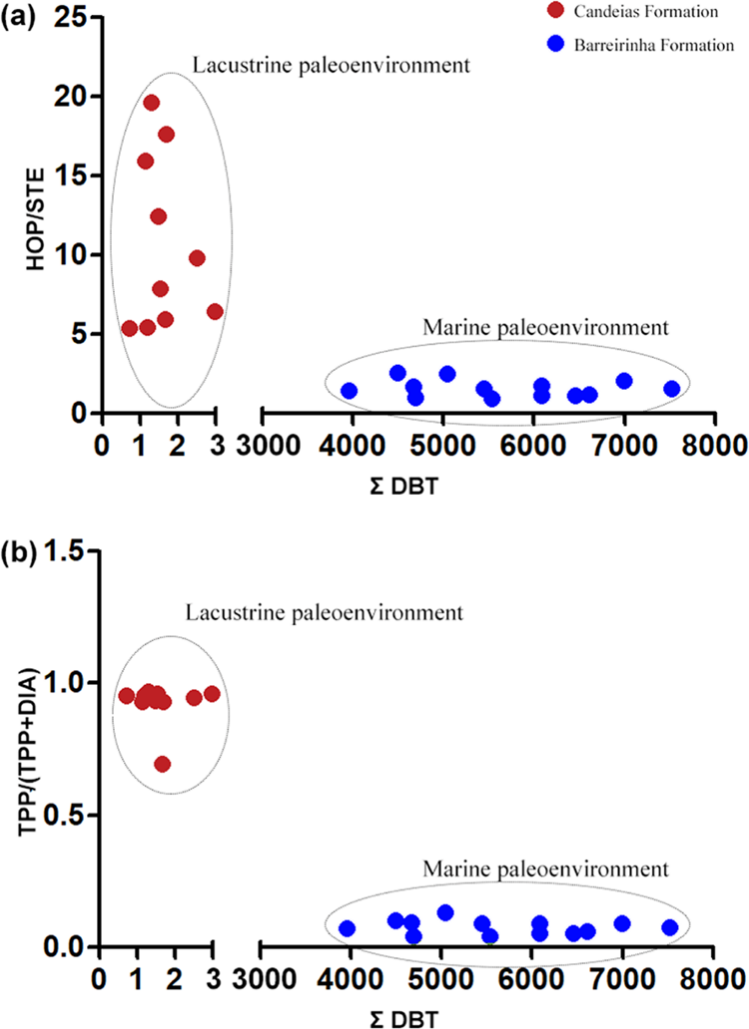
Relationships between: (a) dibenzothiophenes (∑DBT) and the hopanes/steranes (HOP/EST) ratio and (b) TPP/TPP + DIA ratio of samples from the Candeias Formation (Recôncavo basin) and Barreirinha Formation (Amazon basin). Red dotted points are Candeias Formation. Blue dotted points are the Barreirinha Formation.

The relationships between ∑DBT versus HOP/STE (Figure 7a) and TPP/[TPP + DIA] (Figure 7b) showed a good distinction between the depositional paleoenvironments. Samples from the Candeias Formation presented the lowest concentrations of ∑DBT and highest values for HOP/STE and TPP/[TPP + DIA] ratios, which are the characteristics of a freshwater lacustrine paleoenvironment.^10,13^ Samples from the Barreirinha Formation presented the highest concentrations of DBT and lower values for the HOP/STE and TPP/[TPP + DIA] ratios, typical of a marine paleoenvironment.

According to the theory of bacterial sulfate reduction, organosulfur compounds are formed from the production of inorganic sulfur by bacteria during diagenesis.^1^ In general, samples from the Candeias Formation show indications of a high microbial contribution (high values for the HOP/STE ratio) and low concentrations of organosulfur compounds. In this case, the low S-markers concentrations are due to the low availability of sulfate ions in the freshwater lacustrine environment.^48^

**Figure 8:**
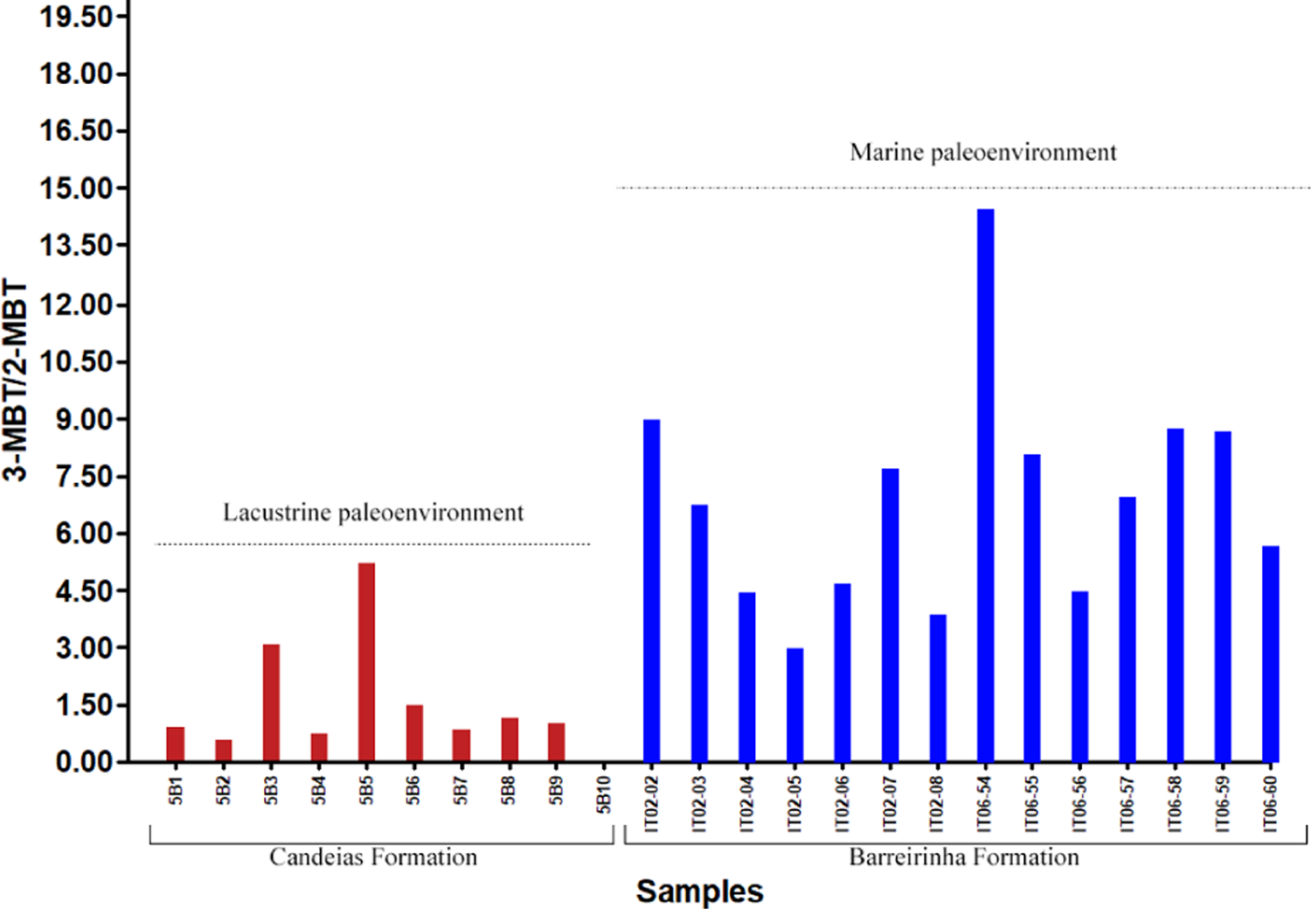
3-MBT/2-MBT ratio for depositional paleoenvironment interpretations of samples from the Candeias Formation (Recôncavo basin) and Barreirinha Formation (Amazon basin).

The BT class distribution can be applied to evaluate differences in source material/depositional paleoenvironment and/or maturity.^47^ All samples analyzed in this study are thermally immature (as will be discussed in subsection 4.5); thus, the differences in BT and alkyl-BT concentrations reflect different depositional paleoenvironments. For example, the 3-MBT/2-MBT ratio allows close inspection of the data to evaluate crude oils from different paleoenvironments.^12,49^ The application of this ratio (Figure 8) in samples indicates the predominance of the lacustrine paleoenvironment in the Candeias Formation (values ≤ 5.22, average of 1.50) and marine paleoenvironment in the Barreirinha Formation (values ≤ 14.42, average of 6.88).

In the outcrops, vertical variations in the concentrations of BTs and, consequently, in the values of the 3-MBT/2-MBT ratio are observed. Such variations may reflect changes over the time of sediment deposition of the geological formations in question. However, this fact has not been completely understood since no correlations were observed between the concentrations of BTs and other geochemical parameters, such as TOC, Rock-Eval pyrolysis data, or saturated biomarkers data.

**Figure 9:**
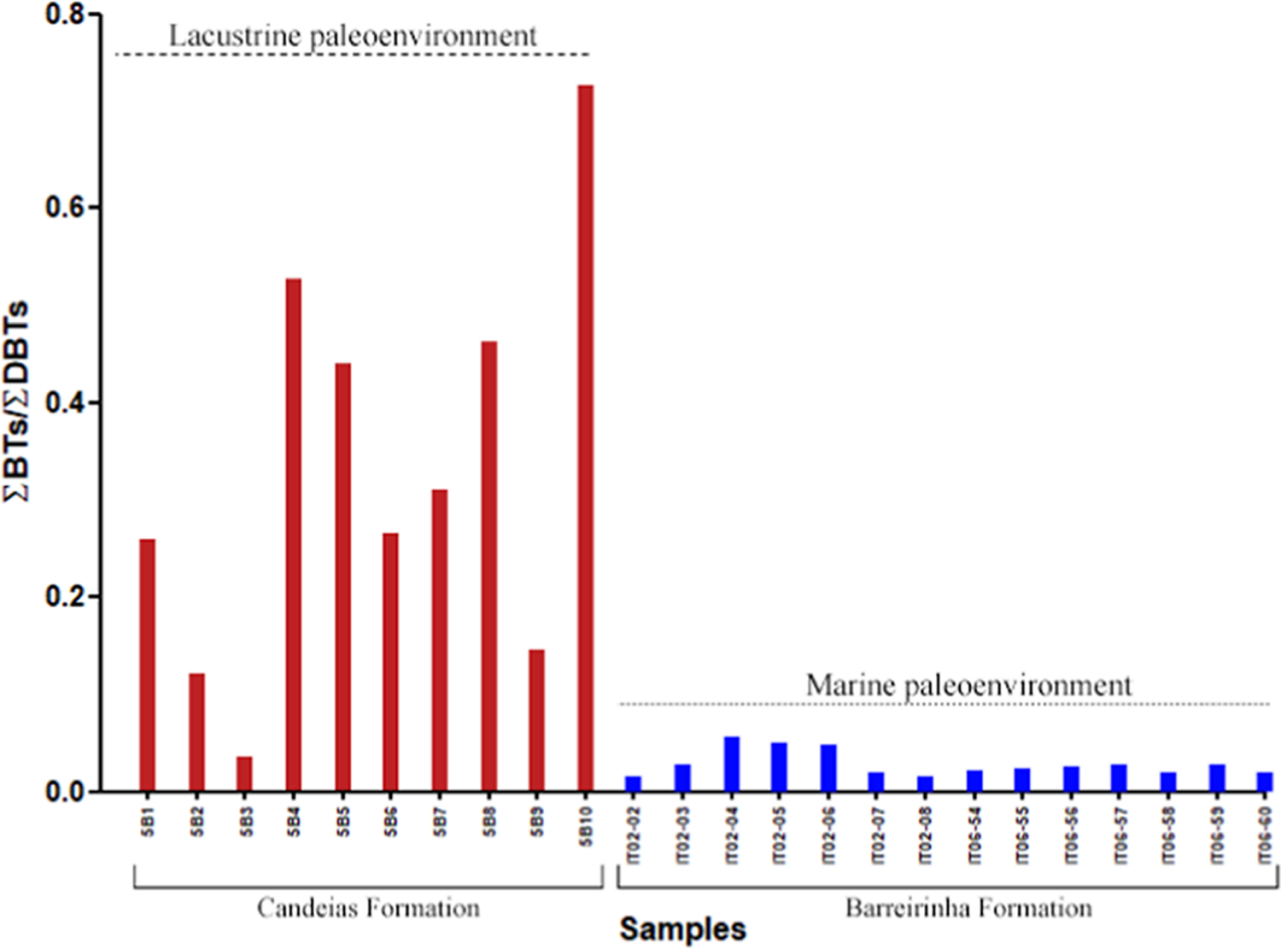
∑BTs/∑DBTs ratio for depositional paleoenvironment interpretations of samples from the Candeias Formation (Recôncavo basin) and Barreirinha Formation (Amazon basin).

BTs/DBTs ratio >3 indicates marine paleoenvironments, from 1 to 3 marine or lacustrine paleoenvironments, and <1 suggests lacustrine paleoenvironments.^8^ DBTs are predominant over BTs for the entire set of samples studied (Figure 6). However, the application of the ∑BTs/∑DBTs ratio to the samples (Figure 9) exhibits contradictory values to those reported previously,^8^ with marine samples showing the lowest values (<0.2). This behavior can be explained by the influence of the depositional paleoenvironment on these compounds, where higher concentrations of DBTs result in lower values in the ∑BTs/∑DBTs ratio in samples from marine paleoenvironment (Barreirinha Formation). Thermochemical sulfate reduction (TSR) associated with relatively high temperatures between 100 and 180 °C can be the abiotic alteration process responsible for the high DBT values observed in the Barreirinha Formation samples.

**Figure 10:**
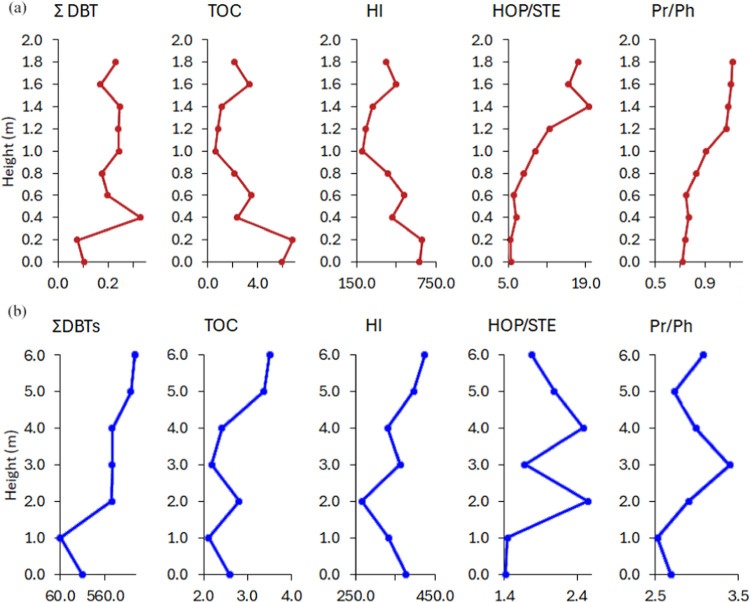
Vertical variations to ∑BNT, TOC, HI, HOP/STE, and Pr/Ph ratios from the (a) Candeias Formation and (b) Barreirinha Formation. For individual values, see Table S2.

The variations in ∑DBT and TOC in the outcrop of the Candeias Formation show an inversely proportional relationship, while in the Barreirinha Formation, there is a proportional increase in DBT and TOC (Figure 10a,b). These results could indicate that the source of organic matter exerts some control over the distribution of DBTs. On the other hand, in the evaluation of the ∑DBT with the geochemical parameters such as hydrogen index (HI), HOP/STE, and Pr/Ph the vertical variations do not agree. Thus, there is uncertainty as to whether the DBT is predominantly controlled by environmental, source, or both factors.

### 4.3 Input of Organic Matter

The evaluation of organic matter input by S-markers can amplify the interpretations generated from saturated biomarkers already extensively described in the literature. Therefore, combining S-markers with biomarkers and other geochemical data allows for increased interpretations of organic matter input into depositional environments.

**Figure 11:**
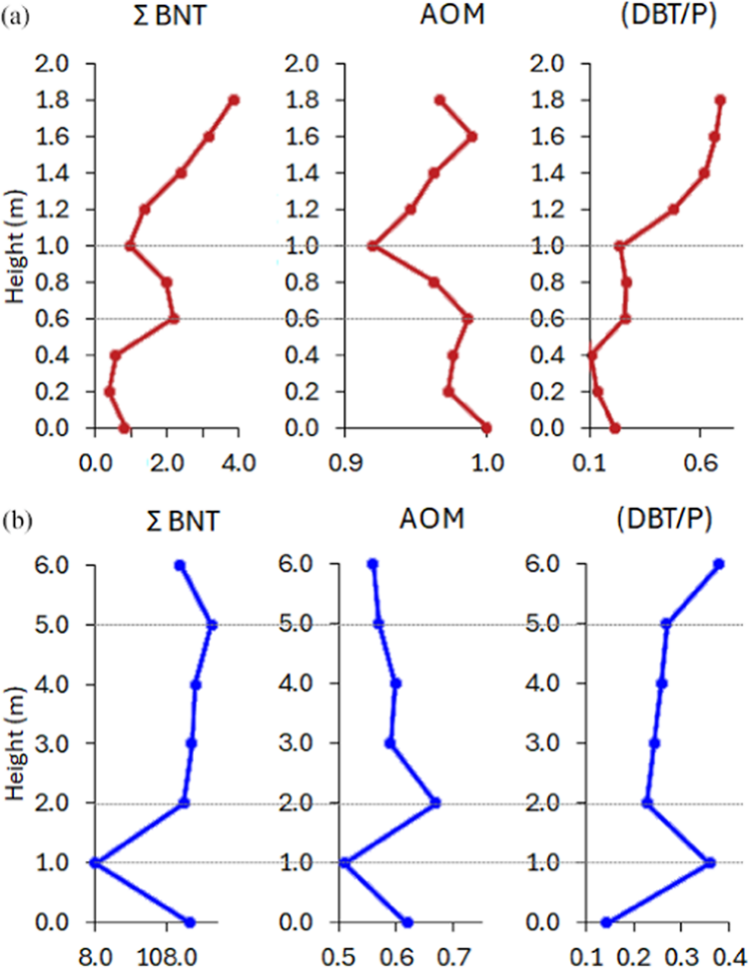
Vertical variations to BNTs, AOM, and DBT/Phen ratios from (a) Candeias Formation and (b) Barreirinha Formation.

Although BNTs have no confirmed origin, a previous study carried out under aerobic conditions indicated that they could be microbially produced from BNT with *Pseudomonas*.^50^ Recent studies have affirmed the possibility that BNTs originate from microbial action.^51−53^ In the evaluation of the vertical variations in two outcrops (Figure 11), there is a similarity in the distribution of the sum of BNTs to the amounts of AOM, which is congruent with the idea that BNTs can come from microbial activity. Furthermore, the DBT/Phen ratio, which can provide information about the *E*_h_ conditions of depositional paleoenvironments,^10^ shows different behavior in the outcrops (Table 3). For the Candeias Formation (Figure 11a), the DBT/Phen ratio varies proportionally with the BNTs and AOM values. In contrast, for the Barreirinha Formation (Figure 11b), the DBT/Phen ratio varies inversely with the other factors under analysis. This behavior may be indicative that BNTs are generated from the AOM under varying *E*_h_ conditions (anoxic for the Candeias Formation and suboxic for the Barreirinha Formation).

**Table 3:** Classification of Depositional Paleoenvironments According to the Redox Condition

zone	redox conditions	[Fe]/[S]	Pr/Ph	DBT/Phen
1A, 1B	anoxic/sulfidic^a^	[Fe] < [S]	<1	>1
2	anoxic/fermentative	[Fe] > [S]	<1	<1
1A, 1B, 2	anoxic/hypersaline	variable	<0.4	variable
3	anoxic/nonsulfidic^a^	[Fe] > [S]	1–3	<1
4	periodically oxic or dysoxic	[Fe] ≫ [S]	>3	<1

a The term sulfidic refers to conditions where free H_2_S*_n_* species are present. [Fe] represents the concentration of iron capable of reacting with reduced sulfur to form iron sulfides. [S] represents the concentration of reduced sulfur capable of reacting with iron to form iron sulfides. Adapted from Hughes et al.^10^

**Figure 12:**
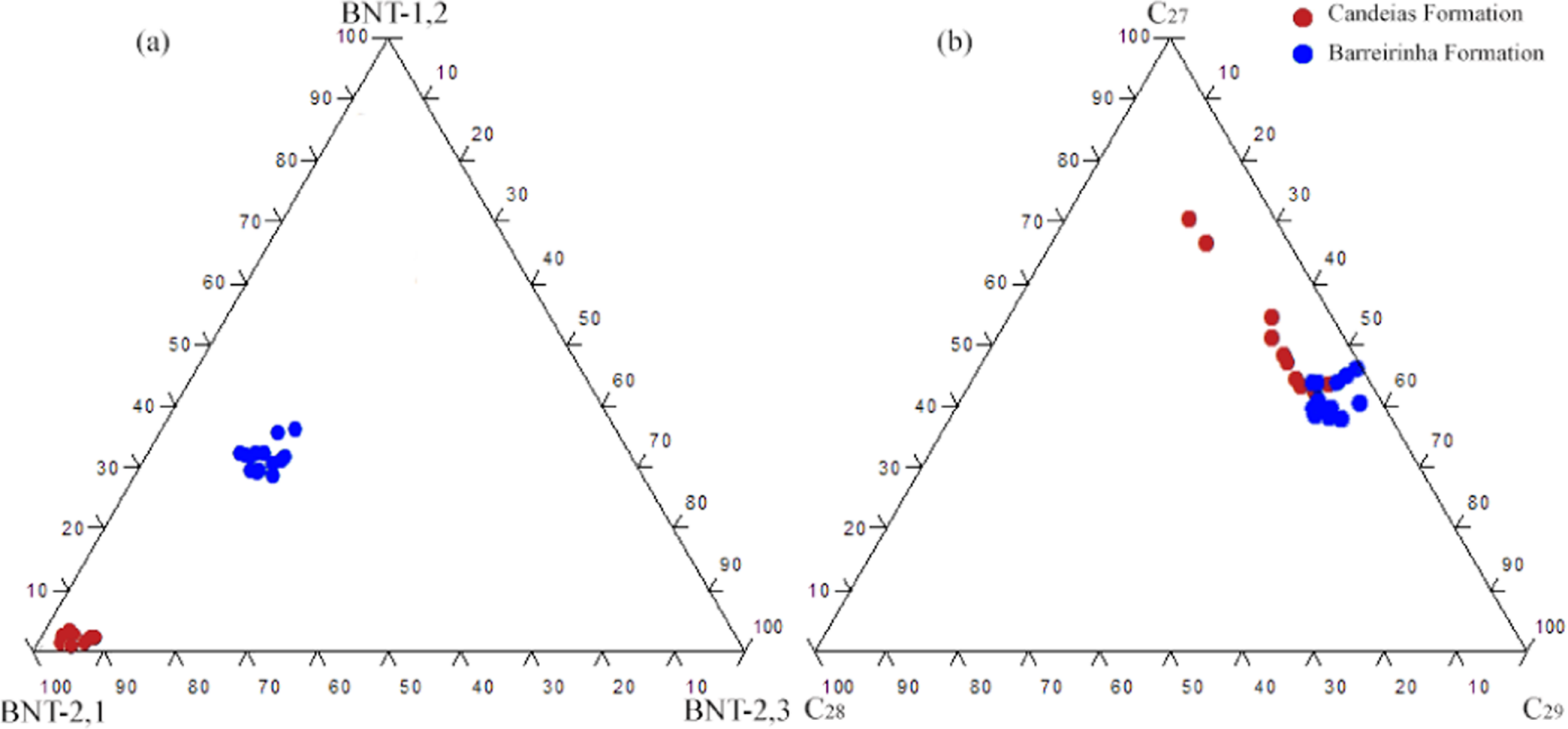
Ternary diagrams for input evaluation. (a) Isomeric distribution of benzothiophenes and (b) distribution of regular steranes C_27_, C_28_, and C_29_ for samples.

In this sense, the abundance of BNTs suggests a microbial contribution to the source rocks that expelled the oils. The concentrations of BNTs in samples are very different (Figure 6), and their relative proportions allow the assessment of the type of organic matter in the depositional paleoenvironment. The BNT concentrations indicate diverse microbial participation in the alteration of the samples. The ternary diagram (Figure 12a) shows the BNTs isomers with the distribution of samples into two groups. In addition, the chart from regular steranes C_27_, C_28_, and C_29_ (Figure 12b) indicates the presence of different organic matter inputs and algae inputs (relative proportion of C_27_ sterane). The C_29_ concentration in samples indicate terrestrial material imputs.^54,55^

The C_27_/C_29_ steranes ratio (Figure 12b) indicates inputs of algal and terrigenous material predominant in the Candeias and Barreirinha Formations, respectively. However, the results of the relative proportions of BNT (Figure 12a) indicate an isomeric differentiation among the samples under study. The benzo[*b*]naphtho[2,1-*d*]thiophene isomer is present in higher concentrations in samples from the Candeias Formation of freshwater lacustrine origin (Figure 12a). The predominance of the benzo[*b*]naphtho[1,2-*d*]thiophene isomer is noted in the Barreirinha Formation, of marine origin (Figure 12a). This observation indicates that the isomeric distribution of BNTs has the potential to distinguish marine and nonmarine depositional environments. Therefore, the benzonaphthothiophenes isomers differences may be associated with different origins (marine or lacustrine) or different oxygenation conditions in depositional paleoenvironments, a fact that alters microbial production^56^

### 4.4 Depositional Paleoenvironment Conditions

**Figure 13:**
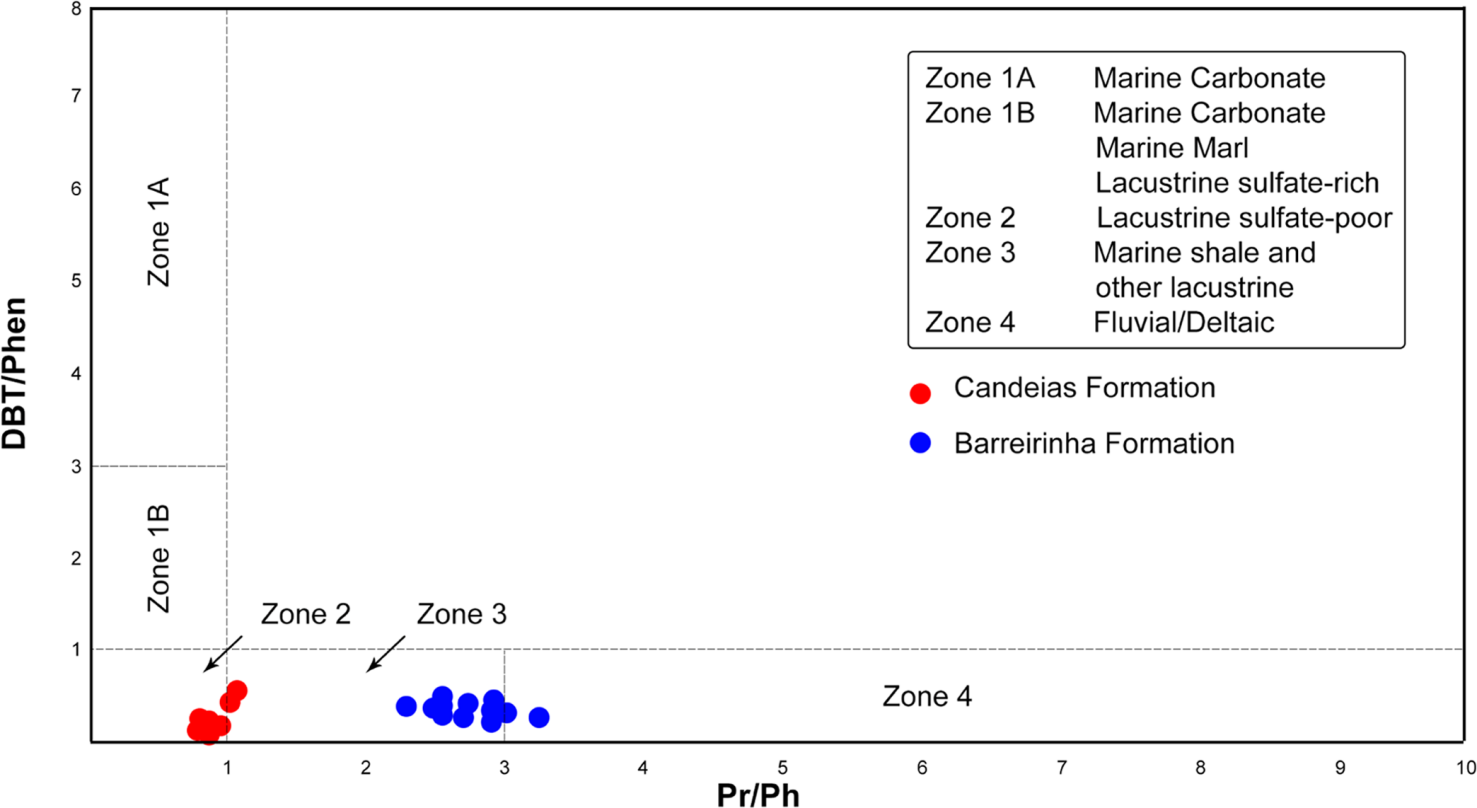
Cross plot of pristane/phytane (Pr/Ph) vs dibenzothiophene/phenanthrene (DBT/PHEN). Red dot points are Candeias Formation. Blue dot points are Barreirinha Formation.

The DBT/Phen ratio, together with the Pr/Ph ratio (Figure 13), provides a powerful way to classify source rock depositional paleoenvironments relative to their most important microbiological and chemical processes.^10^ Applying the relationship between the DBT/Phen and Pr/Ph ratios for the samples from both Formations (Candeias and Barreirinha) indicates zones 2 and 3 as defined by Hughes et al.^10^ These zones distinguish the depositional paleoenvironments of the samples in studies compatible with the interpretations already made: lacustrine paleoenvironment for samples from the Candeias Formation and marine paleoenvironment for samples from the Barreirinha Formation.

**Figure 14:**
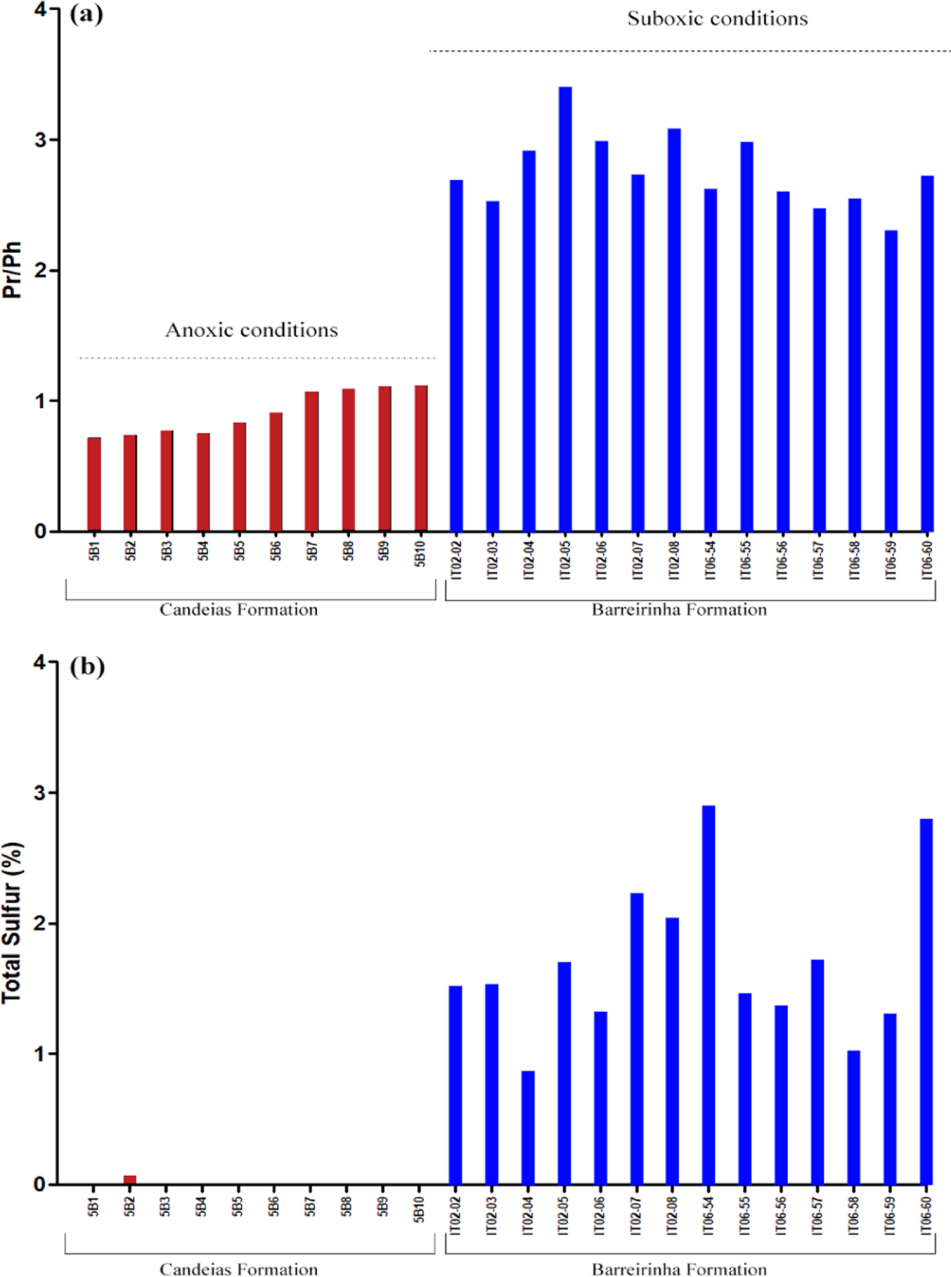
(a) values for the pristane/phytane ratio (Pr/Ph) and (b) total sulfur for the samples of Candeias and Barreirinha Formations applied to evaluate the depositional paleoenvironmental conditions of these formations. For individual values, see Table S2.

The isoprenoids Pr/Ph ratio indicates oxidizing (Pr/Ph > 1) or reducing (Pr/Ph < 1) conditions of the depositional paleoenvironment of the organic matter.^13^ In Figure 14a, the differences between the samples from the Candeias Formation (deposited under more reducing conditions) and the Barreirinha Formation (deposited under more oxidizing conditions) confirm the previous depositional conditions observed.

Variations in the values for the Pr/Ph ratio can be noted in the vertical sections of the outcrops (Figure 14a). In the Candeias Formation, values grade linearly from base to top, with 0.72 at the base and 1.12 at the top. In the outcrops of the Barreirinha Formation, greater vertical variations are observed with values varying between 2.30 and 3.40 (Table S2). Although values greater than 1 were noted in all outcrops studied, due to the good preservation of organic matter, depositional paleoenvironments with these high values were interpreted as presenting suboxic conditions.

The DBT/Phen ratio also reflects the availability of reactive sulfur, primarily hydrogen sulfide (H_2_S), and polysulfides (H_2_S*_n_*), for interaction with organic matter.^10^ Candeias and Barreirinha Formations had low values for this ratio (Figure 14b).

Deposited in a freshwater lacustrine context, samples from the Candeias Formation present low values for the DBT/Phen ratio (Table S3) due to the sulfate concentration in freshwater that varies between ∼10 and 500 μM, which is many times lower than in seawater (28 mM).^57^ In this case, the low *E*_h_ may be caused by fermentation and not sulfate reduction.^48^ Therefore, Pr/Ph < 1 and DBT/Phen <1 may be due to fermentation under low sulfate concentrations.

Barreirinha Formation samples have high values of total sulfur (Figure 14b), originating from the reduction of sulfate present in the marine paleoenvironment. However, the DBT/Phen ratio values for these samples are low (Table S3). This behavior is similar to a previous study with crude oils, where levels of organic sulfides were higher than thiophenes.^58^ Furthermore, Barreirinha Formation source rocks were deposited under conditions of sulfate reduction, and the supply of reactive iron was greater than that of the sulfide. The presence of pyrite in samples from the Barreirinha Formation is geological evidence of this process.^23^

The DBT/Phen values for the outcrops remain between 0 and 1 along the vertical sections, varying between 0.06 and 0.64 in the Candeias Formation and between 0.14 and 0.44 in the Barreirinha Formation. Therefore, it is possible to classify the samples in zones 2 (Candeias Formation) and 3 (Barreirinha Formation), according to the proposed by Hughes et al.^10^ (Table 4).

**Table 4:** Diagnostic Ratios of Sulfur Markers and Biomarkers Used to Evaluate Thermal Maturation in the Outcrop Samples

	Candeias formation									
ratio	5B01	5B02	5B03	5B04	5B05	5B06	5B07	5B08	5B09	5B10
4,6-DMDBT/2,4,6-TMDBT	0.35	0.57	0.45	0.25	0.34	0.43	0.34	0.34	0.32	0.55
2,4,6-TMDBT/2,4,7 + 2,4,8-TMDBT	0.43	0.42	0.68	0.56	0.54	0.72	0.76	0.69	1.01	0.65
*T*_s_/*T*_s_ + *T*_m_	0.03	0.05	0.02	0.02	0.05	0.02	0.02	0.01	0.03	0.04
*T*_max_ (°C)	434	437	438	438	436	412	414	416	415	415

Barreirinha formation														
	IT02-02	IT02-03	IT02-04	IT02-05	IT02-06	IT02-07	IT02-08	IT06-54	IT06-55	IT06-56	IT06-57	IT06-58	IT06-59	IT06-60
ratio	0.89	1.21	1.13	1.16	1.00	1.07	5.37	1.07	0.99	1.17	1.22	1.08	1.16	1.03
4,6-DMDBT/2,4,6-TMDBT	0.34	0.35	0.37	0.34	0.34	0.34	0.32	0.38	0.32	0.35	0.38	0.37	0.35	0.36
2,4,6-TMDBT/2,4,7 + 2,4,8-TMDBT	0.16	0.17	0.16	0.13	0.14	0.15	0.17	0.15	0.16	0.15	0.17	0.02	0.14	0.17
*T*_s_/*T*_s_ + *T*_m_	435	436	433	437	436	434	433	426	434	434	428	427	429	425
*T*_max_ (°C)														

### 4.5 Thermal Maturation

Sulfur compounds are good markers of the thermal maturation stage due to their highest stability at elevated temperatures.^8,9^ Generally, the concentrations of BTs, DBTs, and BNTs increase with thermal maturation. Diagnostic ratios such as 4,6-DMDBT/2,4,6-TMDBT and 2,4,6-TMDBT/(2,4,7 + 2,4,8)-TMDBT are employed to evaluate this parameter.^1^

The low values of the S-markers ratios (Table 2) for all samples analyzed indicated that they are thermally immature, in accordance with the interpretations reported in the literature for these compounds.^49,59^ In addition, low values of the ratio *T*_s_/(*T*_s_ + *T*_m_) and *T*_max_ values less than 440 °C confirm the thermal immaturity of the samples for hydrocarbon generation.^13^

In addition, there is a correlation between the maturation values tested in kerogen samples (evaluated by *T*_max_ from Rock-Eval pyrolysis) and the values calculated from molecular parameters. For the entire set of samples, the *T*_max_ values are low (less than 440 °C), ranging between 412 and 438 °C for the samples from the Candeias Formation and between 425 and 437 °C for the samples from the Barreirinha Formation. The ratio of biomarkers *T*_s_/(*T*_s_ + *T*_m_) also shows low values for the samples under study, attesting to thermal immaturity of the samples for hydrocarbon generation.^13^

Although they allow the same interpretation regarding thermal immaturity, the values calculated from the molecular ratios are not directly proportional to the *T*_max_ values, indicating that both analyses must be performed in such a way that they corroborate the other.

## 5 Conclusions

The application of GC–MS/MS instead of GC–MS for individual quantification of twenty-one S-markers allowed an increase in the reliability and accuracy of data results. The results obtained by S-markers allowed us to evaluate and prove distinctions and similarities between the source rocks, defining the origin of organic matter, the organic matter input, the depositional paleoenvironment conditions, and the level of thermal maturity of the samples.

The samples from the Recôncavo basin, of lacustrine origin, have lower concentrations of BT, DBT, and BNT, while samples from the Amazon basin, of marine origin, present high concentrations of these compounds. There is an inversely proportional relationship between variations in ∑DBT and TOC in the outcrop of the Candeias Formation, while in the Barreirinha Formation, this proportion increases. This observation is indicative that the source of organic matter exerts some control of the distribution of DBTs.

The relative proportions of the BNT-2,1, BNT-1,2, and BNT-2,3 isomers, in addition to the C_27_, C_28_, and C_29_ regular steranes, indicated that the inputs of algal and terrigenous materials were predominant in the Candeias and Barreirinha Formations, respectively, with different levels of microbial participation. In addition, in the vertical variations of the outcrops, there is a similarity in the distribution of the sum of BNTs with the amounts of AOM, which indicates that BNTs can come from microbial activity.

Similarities between DBT/Phen concentrations and Pr/Ph values indicated that S-markers are affected by the conditions of the depositional paleoenvironments. Candeias Formation presented low values for the DBT/Phen and Pr/Ph ratios due to the absence of sulfate in the paleodepositional environment and the fermentation of organic matter. Although the source rocks of the Barreirinha Formation were formed under conditions of sulfate reduction, the supply of reactive iron exceeded that of sulfide, resulting in the predominant formation of pyrite.

The thermal maturity was evaluated using *T*_max_ values from Rock-Eval pyrolysis from kerogen samples, and the molecular values were calculated using S-markers. Low values to ratios 4,6-DMDBT/2,4,6-TMDBT and 2,4,6-TMDBT/(2,4,7 + 2,4,8)-TMDBT associated with low values to *T*_max_ (less than 440 °C) indicated that the Formations are thermally immature for hydrocarbon generation.

## Supporting Information Available

Palynofacies data, saturated biomarkers, and BNTs concentrations in samples (Table S1); TOC, total sulfur, Rock-Eval pyrolysis parameters (Table S2); diagnostic ratios and concentration of saturated and S-markers in samples (Table S3); GC–MS chromatograms of the biomarkers in saturated fractions (Figures S1–S4) (PDF)
