# Supplementary material for: Organic Sulfur Markers as Proxies of Depositional Paleoeenvironments Related to Recôncavo and Amazon Basins, Brazil
Source: ACS Omega. 2024 Nov 1;9(45):45358–75. doi: 10.1021/acsomega.4c07344 (PMC11561611; doi:10.1021/acsomega.4c07344)
Supplement: Supplementary file 1 — ao4c07344_si_001.pdf [file ao4c07344_si_001.pdf]

ORGANIC SULFUR MARKERS AS PROXIES OF DEPOSITIONAL PALEOENVIRONMENTS RELATED TO RECÔNCAVO AND AMAZON BASINS, BRAZIL

Diego Nery do Amaral[1,4], Flávia Lima e Cima Miranda[1], Lua Morena Leôncio de Oliveira[1], José Roberto Cerqueira[1], Hélio Jorge Portugal Severiano Ribeiro[5], Olívia Maria Cordeiro Oliveira[1], Antônio Fernando de Souza Queiroz[1], Sérgio Luís Costa Ferreira[1,2] Maria Elisabete Machado[1,2,3*]

[1] Universidade Federal da Bahia, Programa de Pós-Graduação em Petróleo e Meio Ambiente, 40170-115, Salvador, BA, Brazil

[2] Universidade Federal da Bahia, Instituto de Química, 40170-115, Salvador, BA, Brazil

[3] Instituto Nacional de Energia e Ambiente, INCT&EIA, 40170-115, Salvador, BA, Brazil

[4] Departamento de Ciências Exatas, Universidade Estadual de Feira de Santana, BA, Brazil

[5] Laboratório de Engenharia e Exploração de Petróleo, LENEP, 27930-480, Macaé, RJ, Brazil

*corresponding author: e-*mail address*: maria.elisabete@ufba.br

Table S1- Palynofacies data, saturated biomarkers and proportions of BNTs from the samples

Sample	AOM (%)	Palynomorphs (%)	Phytoclats (%)	HOP/STE	TPP/TPP+DIA	C_{27} sterane (%)	C_{28} sterane (%)	C_{29} sterane (%)	BNT-2,1 (%)	BNT-1,2 (%)	BNT-2,3 (%)	Σ BNT
5B1	95	1.33	3.66	5.48	0.95	48.6	2.87	48.6	95.3	2.75	2.02	0.83
5B2	92.3	5.00	2.60	5.41	0.95	48.4	2.98	48.6	96.0	1.68	2.27	0.40
5B3	92.7	2.66	4.66	6.45	0.96	50.7	3.01	46.3	94.1	3.32	2.56	0.58
5B4	93.7	3.66	2.68	5.95	0.69	52.0	2.94	45.0	94.6	2.28	3.08	2.19
5B5	91.3	4.00	4.66	7.86	0.96	55.0	2.92	42.0	93.4	2.70	3.88	1.98
5B6	87.0	5.00	8.00	9.84	0.95	56.0	3.02	41.0	91.7	2.41	5.87	0.97
5B7	90.0	3.66	6.34	12.43	0.93	59.4	2.65	37.9	90.8	2.40	6.77	1.37
5B8	91.3	2.66	6.01	19.65	0.97	88.1	4.12	7.7	92.7	1.53	5.79	2.40
5B9	94.7	3.00	2.33	15.95	0.93	61.2	2.62	36.2	95.2	0.76	4.05	3.16
5B10	91.7	4.33	4.01	17.62	0.93	91.3	3.71	4.9	93.7	2.03	4.24	3.86
IT02-02	57.0	40.0	4.00	1.42	0.07	39.4	8.48	52.1	55.0	31.9	13.1	141
IT02-03	46.0	50.0	3.00	1.45	0.08	39.6	10.3	50.1	55.5	29.8	14.7	8.18
IT02-04	62.0	34.0	3.00	2.55	0.10	38.0	7.17	54.8	54.5	29.2	16.3	131
IT02-05	54.0	43.0	2.00	1.68	0.09	39.7	7.91	52.4	50.4	31.1	18.5	143
IT02-06	55.0	41.0	3.00	2.49	0.13	38.4	8.77	52.8	49.5	31.7	18.7	148
IT02-07	52.0	47.0	1.00	2.09	0.09	38.5	10.4	51.1	45.8	36.3	17.9	171
IT02-08	51.0	47.0	1.00	1.78	0.09	43.6	7.62	48.8	48.4	35.7	15.8	127
IT06-54	66.0	33.0	1.00	1.56	0.08	40.6	9.06	50.3	53.3	32.1	14.5	173
IT06-55	69.0	29.0	3.00	1.19	0.06	43.7	8.3	47.9	54.2	31.8	14.0	168
IT06-56	59.0	38.0	2.00	1.11	0.05	44.1	8.02	47.9	55.5	32.3	12.2	163
IT06-57	67.0	31.0	2.00	0.94	0.04	45.8	1.41	52.8	54.2	29.7	16.1	167
IT06-58	63.0	35.0	2.00	1.04	0.04	40.6	3.2	56.2	52.6	28.7	18.6	162
IT06-59	67.0	32.0	3.00	1.13	0.05	43.9	4.67	51.4	51.7	30.6	17.6	159
IT06-60	60.0	36.0	3.00	1.58	0.09	45.0	3.24	51.8	52.2	32.3	15.5	149

Table S2 - TOC, total sulfur, Rock-Eval pyrolysis parameters and Pr/Ph ratio of the samples.

Sample	TOC (%)	IR (%)	Sulfur (%)	(S1) (mg/g)	(S2) (mg/g)	(S3) (mg/g)	Tmax (ºC)	HI	OI	PI	Pr/Ph
5B1	5.91	74.2	<0,05	0.35	27.1	0.48	434	625	2	0.013	0.72
5B2	6.77	71.4	0.07	0.47	29.9	0.35	437	646	8	0.015	0.74
5B3	2.35	72.3	<0,05	0.12	7.48	0.07	438	418	4	0.016	0.77
5B4	3.51	75.2	<0,05	0.21	14.1	0.11	438	511	4	0.015	0.75
5B5	2.15	75.5	<0,05	0.11	7.07	0.1	436	386	5	0.015	0.83
5B6	0.63	91.0	<0,05	0.04	1.41	0.05	412	193	7	0.028	0.91
5B7	0.83	84.3	<0,05	0.05	2.1	0.1	414	221	11	0.023	1.07
5B8	1.12	82.5	<0,05	0.08	3.3	0.09	416	270	7	0.024	1.09
5B9	3.32	79.9	<0,05	0.19	9.66	0.25	415	451	12	0.019	1.11
5B10	2.16	78.9	<0,05	0.09	5.36	0.12	415	375	8	0.017	1.12
IT02-02	2.59	99.9	1.52	0.15	9.77	0.1	435	377	5	0.015	2.69
IT02-03	2.11	99.9	1.53	0.12	7.3	0.18	436	333	8	0.016	2.53
IT02-04	2.81	99.9	0.87	0.13	7.55	0.26	433	266	9	0.017	2.91
IT02-05	2.19	99.9	1.7	0.13	8.1	0.1	437	363	4	0.016	3.40
IT02-06	2.42	99.9	1.32	0.12	8.28	0.12	436	331	5	0.014	2.99
IT02-07	3.38	99.9	2.23	0.21	13.3	0.13	434	396	4	0.016	2.73
IT02-08	3.51	99.9	2.04	0.26	14.7	0.13	433	424	4	0.017	3.08
IT06-54	5.14	99.9	2.9	0.33	10.2	0.18	426	270	5	0.031	2.62
IT06-55	4.82	99.9	1.46	0.31	20.0	0.82	434	444	18	0.015	2.98
IT06-56	5.56	99.9	1.37	0.47	26.7	1.07	434	532	21	0.017	2.60
IT06-57	3.88	99.9	1.72	0.29	11.9	0.19	428	326	5	0.024	2.47
IT06-58	3.86	99.9	1.02	0.2	10.7	0.24	427	291	7	0.018	2.55
IT06-59	3.36	99.9	1.31	0.22	11.1	0.31	429	346	10	0.02	2.30
IT06-60	3.7	99.9	2.8	0.29	10.6	0.05	425	305	1	0.027	2.72

Table S3 – Ratios using S-markers and Ts/Ts+Tm ratio of the samples.

Sample	3-MBT/2-MBT	ΣBTs/ΣDBTs	DBT/PHEN	4,6-DMDBT/2,4,6-TMDBT	2,4,6-TMDBT/(2,4,7 + 2,4,8)-TMDBT	Ts/Ts+Tm
5B1	0.90	0.26	0.16	0.35	0.43	0.03
5B2	0.59	0.12	0.08	0.57	0.42	0.05
5B3	3.08	0.04	0.06	0.45	0.68	0.02
5B4	0.74	0.53	0.21	0.25	0.56	0.02
5B5	5.22	0.44	0.22	0.34	0.54	0.05
5B6	1.48	0.27	0.19	0.43	0.72	0.02
5B7	0.85	0.31	0.43	0.34	0.76	0.02
5B8	1.15	0.46	0.57	0.34	0.69	0.01
5B9	1.00	0.15	0.62	0.32	1.01	0.03
5B10	0.03	0.73	0.64	0.55	0.65	0.04
IT02-02	8.98	0.02	0.14	0.89	0.34	0.16
IT02-03	6.73	0.03	0.36	1.21	0.35	0.17
IT02-04	4.43	0.06	0.23	1.13	0.37	0.16
IT02-05	2.97	0.05	0.24	1.16	0.34	0.13
IT02-06	4.68	0.05	0.26	1.00	0.34	0.14
IT02-07	7.69	0.02	0.27	1.07	0.34	0.15
IT02-08	3.86	0.02	0.38	5.37	0.32	0.17
IT06-54	14.42	0.02	0.43	1.07	0.38	0.15
IT06-55	8.07	0.02	0.44	0.99	0.32	0.16
IT06-56	4.47	0.03	0.39	1.17	0.35	0.15
IT06-57	6.94	0.03	0.34	1.22	0.38	0.17
IT06-58	8.75	0.02	0.29	1.08	0.37	0.02
IT06-59	8.66	0.03	0.38	1.16	0.35	0.14
IT06-60	5.66	0.02	0.30	1.03	0.36	0.17

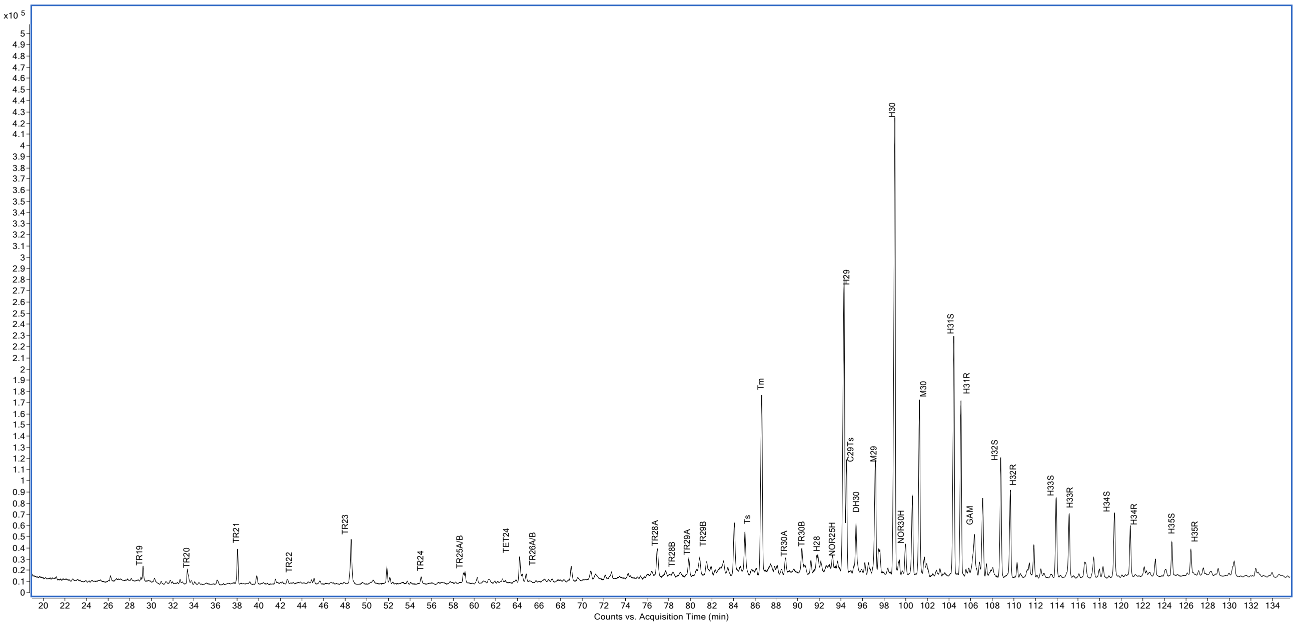

Figure S1 – Representative chromatogram from m\z 191, Barreirinha Formation.

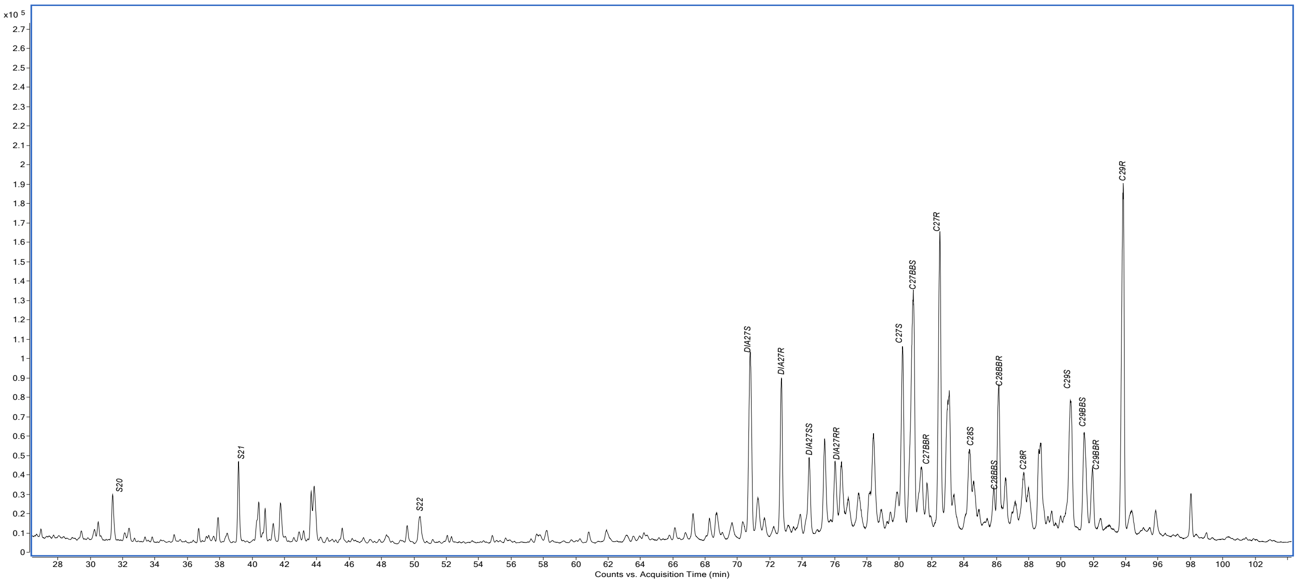

Figure S2 – Representative chromatogram from m\z 217, Barreirinha Formation.

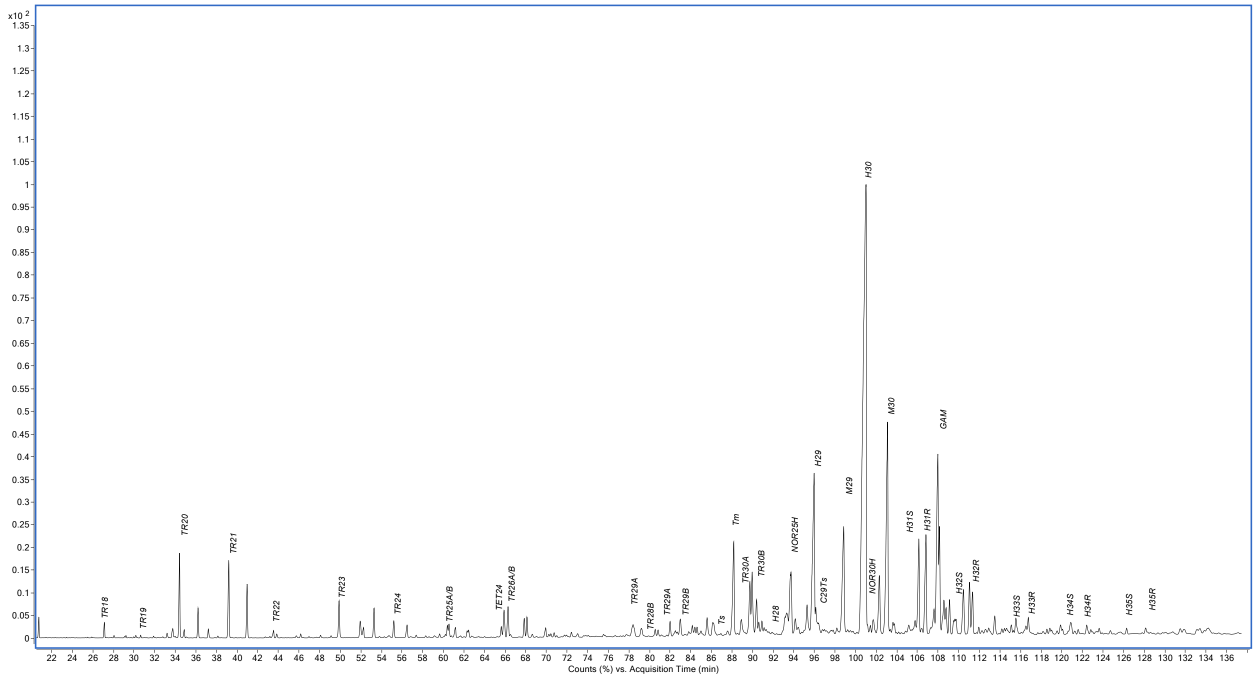

Figure S3 – Representative chromatogram from m\z 191, Candeias Formation.

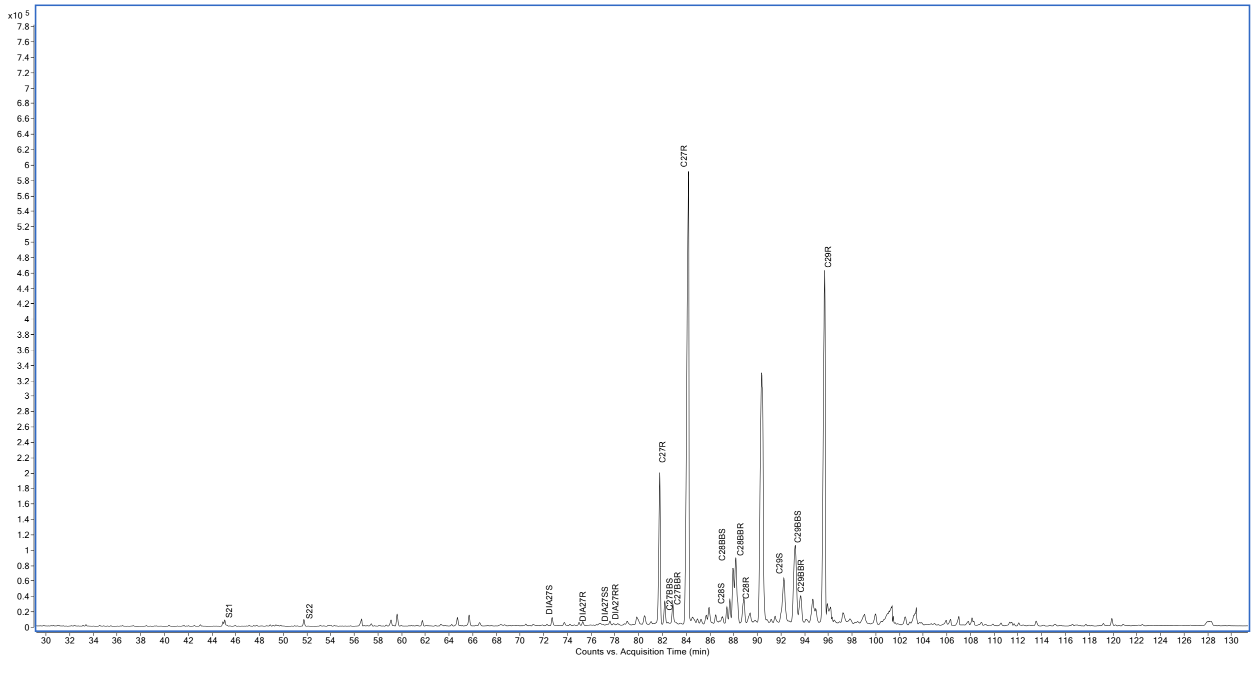

Figure S4 – Representative chromatogram from m\z 217, Candeias Formation.
